# Learning grain boundary segregation energy spectra in polycrystals

**DOI:** 10.1038/s41467-020-20083-6

**Published:** 2020-12-11

**Authors:** Malik Wagih, Peter M. Larsen, Christopher A. Schuh

**Affiliations:** 1 Department of Nuclear Science and Engineering, Massachusetts Institute of Technology, 77 Massachusetts Avenue, Cambridge, MA 02139 USA; 2 Department of Materials Science and Engineering, Massachusetts Institute of Technology, 77 Massachusetts Avenue, Cambridge, MA 02139 USA

**Keywords:** Surfaces, interfaces and thin films, Metals and alloys, Atomistic models, Computational methods

## Abstract

The segregation of solute atoms at grain boundaries (GBs) can profoundly impact the structural properties of metallic alloys, and induce effects that range from strengthening to embrittlement. And, though known to be anisotropic, there is a limited understanding of the variation of solute segregation tendencies across the full, multidimensional GB space, which is critically important in polycrystals where much of that space is represented. Here we develop a machine learning framework that can accurately predict the segregation tendency—quantified by the segregation enthalpy spectrum—of solute atoms at GB sites in polycrystals, based solely on the undecorated (pre-segregation) local atomic environment of such sites. We proceed to use the learning framework to scan across the alloy space, and build an extensive database of segregation energy spectra for more than 250 metal-based binary alloys. The resulting machine learning models and segregation database are key to unlocking the full potential of GB segregation as an alloy design tool, and enable the design of microstructures that maximize the useful impacts of segregation.

## Abstract (web summary)

Predicting segregation energies of alloy systems can be challenging even for a single grain boundary. Here the authors propose a machine-learning framework, which maps the local environments on a distribution of segregation energies, to predict segregation energies of alloy elements in polycrystalline materials.

## Introduction

In alloys, the segregation of solute atoms at grain boundaries (GBs) induces structural effects^1,2^ that include strengthening^3–5^, embrittlement^6,7^, corrosion resistance^8,9^, and GB phase transitions^10,11^. As such, controlling GB segregation is an essential tool for many engineering applications^12^, including, e.g., the thermodynamic stabilization of nanocrystalline alloys against grain growth^13–15^. And, though most technically relevant alloys are used in a polycrystalline form, there is a very limited understanding of GB segregation in polycrystals^16^, and a general lack of databases of segregation information relevant to them.

In a polycrystal, the GB network has a variety of site-types that can either promote or inhibit segregation to different degrees, depending on their unique local atomic environments. The drive for a solute atom to segregate to a GB site-type (i) is quantified by the segregation enthalpy ${\Delta}H_i^{{\rm{seg}}}$, which, in solids^17^, is equivalent to the internal energy difference between the solute atom occupying the GB site, and a bulk (intra-grain) site, ${\Delta}H_i^{{\rm{seg}}} \approx {\Delta}E_i^{{\rm{seg}}} = E_{{\rm{gb}},i}^{{\rm{solute}}} - E_{\rm{c}}^{{\rm{solute}}}$; a negative ${\Delta}E_i^{{\rm{seg}}}$ (i.e. the system reduces energy upon segregation) promotes segregation and vice versa. The spectrum of ${\Delta}E_i^{{\rm{seg}}}$ in a polycrystal will determine the extent of equilibrium GB segregation in an alloy^18–20^. Recently, we have shown this spectrum to be captured by a skew-normal distribution for an Mg solute segregation in an Al polycrystal^20^. However, the computation of these segregation spectra is a resource-intensive task. For example, a (50 nm)^3^ Al polycrystal with an average grain size of 10 nm has roughly one million GB sites, which translates to a million atomistic calculations, where a solute atom is placed substitutionally at each GB site independently and allowed to relax. This makes the task of investigating different microstructures (i.e. multiple polycrystalline samples) cost-prohibitive for a given alloy.

Here, we propose a machine learning (ML) framework that can accurately predict the relaxed segregation energy of a solute atom in a GB site, solely based on its undecorated (pre-segregation) atomic environment. Our approach is tiered and offers two models. The first is a high-fidelity model that is trained to accurately capture the variation of segregation energy across a large swath of the GB space, and thus can be used to study an alloy system in detail and instantaneously evaluate segregation for different microstructures. The second is an accelerated model that uses dimensionality reduction to reproduce the high-fidelity model—with a minimal loss in accuracy—using three orders of magnitude fewer data-points for training (only 100 sites). We use the accelerated approach to scan across the alloy space, and build an extensive database giving GB segregation spectra for all aluminum, magnesium, and transition metals-based binary alloys for which an interatomic potential exists in the Interatomic Potentials Repository^21,22^ of the National Institute of Standards and Technology (NIST) - a total of 259 binary alloys. This database allows us to identify alloys of interest with minimal computational cost, for which high-fidelity models can be trained and used. The proposed ML framework and the resulting spectral segregation database should provide a general and broadly applicable alloy design toolbox relevant to all material properties impacted by solute segregation.

## Results

### High-Fidelity ML model for GB segregation

If a solute atom is substitutionally placed at a GB site and is allowed to relax, its local neighboring atoms will be affected (and possibly displaced) by the introduced elastic and chemical interactions. Hence it follows that the local atomic environment (LAE) of a GB site will influence its favorability for solute segregation, and thus this environment should be accurately captured in any learning model that aims to correlate the undecorated (pre-segregation) GB site to its final decorated (post-segregation) relaxed state. So far, the state-of-the-art learning models in the literature use simple well-known structural features^23,24^, such as atomic volume, coordination, and Voronoi parameters, which mostly limit the description of the LAE to its first nearest-neighbor atoms. Instead, we propose using an atom-centered feature extraction method “descriptor” that encodes the local atomic environment around an atom within a cutoff radius^25,26^. Such descriptors—also known as “fingerprints”—are developed and widely used to construct ML-based interatomic potentials; examples include the atom-centered symmetry functions^27^, bispectrum components^28,29^, and smooth overlap of atomic positions (SOAP)^30^. There are two main advantages to using such atom-centered descriptors. The first is that no a priori knowledge or selection of what constitutes an important structural feature of the LAE (such as volume, coordination, etc.) is required, but rather, by using a complete description of the LAE within a cutoff radius, we relegate the decision of learning the most important features to the ML model. The second is that the use of a large cutoff radius ensures that the most dominant interactions between the solute atom and its LAE are captured. As these descriptors are borrowed from the interatomic potential fitting literature, we can think of our approach as fitting a “pseudo interatomic potential” for solute segregation at GBs.

**Fig. 1:**
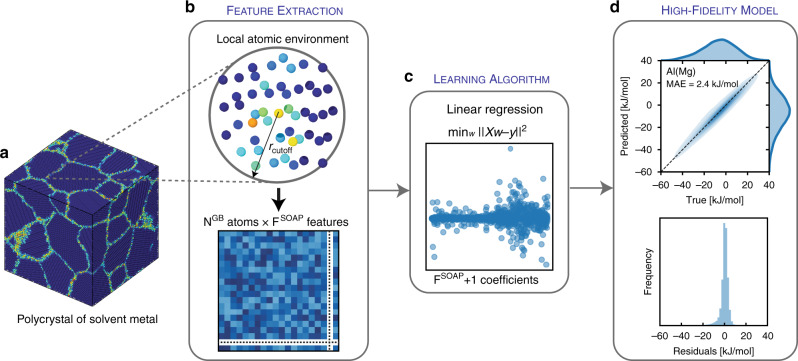
High-fidelity ML model to learn Mg solute^36^ segregation at GBs in an Al polycrystal. For the (**a**) 20x20x20 nm^3^ thermally annealed Al polycrystal with 16 grains (colored by the centro-symmetry parameter^74^), (**b**) the LAE of every identified GB atom is transformed into a feature vector, using the SOAP method^30^ with *r*_cutoff_ = 6 Å, to construct a feature matrix for the full GB network (N^GB^ atoms x F^SOAP^ features), which is used as the input to the (**c**) learning algorithm (linear regression) to learn Mg GB segregation energies, using a 50/50 training/testing split. **d** Predictive performance—mean absolute error (MAE)—of the trained ML model across the full GB network.

The proposed high-fidelity ML model is summarized in Fig. 1, which shows two main steps: (a) feature extraction and (b) a learning algorithm. For feature extraction, we use the SOAP method^30^, as it was recently shown to perform well in describing GB environments (albeit for the different problem of predicting GB energies)^31^. The SOAP method produces for a given GB site and its LAE within a cutoff radius, a feature vector (descriptor) that is invariant under all physical symmetries (permutation, translation, rotation, etc.). The size of the feature vector is controlled by the SOAP hyperparameters (detailed in the methods section), which, in essence, determines the resolution of the vector and its sensitivity to changes in the LAE. In this work, the SOAP feature vector for each GB site has F^SOAP^=1,015 features. For the cutoff radius, we use 6 Å, which is a conservative cutoff used in constructing interatomic potentials, as it captures the most dominant atomic interactions for an atom with its LAE^25,32^. We note that, though we opted to use the same F^SOAP^ and a radial cutoff of 6 Å for all binary alloys (as optimal parameters that require minimal input from the user), this procedure is flexible, and one could, by further optimizing the SOAP hyperparameters to the specific alloy of interest, improve the accuracy of the ML model. (For example, a solute atom that has a large size mismatch with solvent atoms could benefit from a larger radial cutoff.)

The product of the first step of the ML framework, feature extraction, is a feature matrix of size (N^GB^ atoms x F^SOAP^ features), which is used as the input to the second step, the learning algorithm, which learns to map the input SOAP features to the target property (segregation energy). For the learning algorithm, we use linear regression for three reasons: first, it is a simple inexpensive model to train and use for predictions; second, it can be automated as it does not require any hyperparameter optimization; and third, it inherently ensures regularization (i.e. is less prone to overfitting)—by simply using a sample size of >10xF^SOAP^ GB sites (following the “one in ten” rule of thumb^33^, which we further validate in Supplementary Fig. 21) to fit the F + 1 coefficients of the model (F coefficients + intercept), we guard against model overfitting, and selection bias towards a small subset of the population (randomly sampling as few as ~400 points from an infinite population gives a 95% confidence level and 5% margin of error^34^). We note that although more elaborate learning algorithms could be used, such as support vector machines^31^, Gaussian process regression^28^ or neural networks^32^, our proposed ML framework prioritizes simplicity and minimal input from the user, so that other researchers can adopt it easily. We use this approach to showcase the utility of using atom-centered descriptors for learning GB site segregation energies, without getting lost in the intricate details of fine-tuning more advanced learning algorithms. We note that though the proposed learning framework focuses on segregation spectra in substitutional alloys, it is extensible in principle to interstitial alloys by defining interstitial sites^35^ at the GB and bulk regions.

Using the high-fidelity approach, we train a model, in Fig. 1c, for Mg solute^36^ segregation in a thermally annealed 20 × 20 × 20 nm^3^ Al polycrystal that has 16 grains and ~10^5^ GB sites, using a randomized 50/50 split for training/testing. This simple holdout method is easy/cheap to train and use, and its conservative test ratio will guard against a high variance model in most cases. The trained model is highly accurate, with a mean absolute error (MAE) of 2.4(2.5) kJ/mol for the train(test) datasets, respectively, and a root-mean-square error of RMSE=3.8(4.1) kJ/mol. The model faithfully reproduces the distribution of segregation enthalpies in the polycrystal and has a well-behaved error with normally distributed residuals. This result compares favorably with a more sophisticated ML model by Huber et al.^23^, which used 19 structural features (volume, coordination, Voronoi analysis parameters, and Steinhardt bond-order parameters) with gradient boosted decision trees, and had a 9-fold cross-validation RMSE=7.7 kJ/mol for Mg solute segregation in a database of 38 low and high-symmetry boundaries in Al. The comparison is not direct, of course, since that work focused on bi-crystals whereas we use polycrystals, but it is also encouraging that the present error is also much lower than the reported error of the interatomic potential as compared to DFT GB segregation energies, which has an RMSE of 8.7 kJ/mol^24^.

**Fig. 2:**
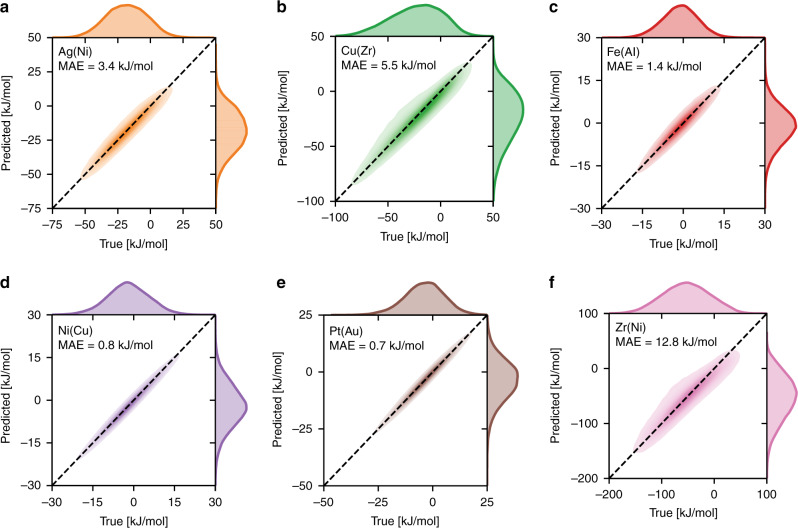
Validation of the high-fidelity ML framework across the alloy space. The predictive performance—mean absolute error (MAE) –of trained ML models, as outlined in Fig. 1, for solute segregation in six 20x20x20 nm^3^ polycrystalline alloys: **a–f** Ag(Ni)^37^, Cu(Zr)^38^, Fe(Al)^39^, Ni(Cu)^40^, Pt(Au)^41^, and Zr(Ni)^42^.

We further validate the efficacy of the high-fidelity ML model for GB solute segregation across the alloy space by training to six more 20 × 20 × 20 nm^3^ polycrystalline volumes for different alloys: Ag(Ni)^37^, Cu(Zr)^38^, Fe(Al)^39^, Ni(Cu)^40^, Pt(Au)^41^, and Zr(Ni)^42^. As shown in Fig. 2, the ML model accurately reproduces the segregation spectra for the six binary alloys, and has a low MAE typically below ~6 kJ/mol and often below 1 kJ/mol. Alloys with higher absolute values (wider distribution) for the segregation energy will correspondingly have a higher MAE, and the worst of these seen here is MAE = 12.8 kJ/mol for the Zr(Ni) system, but here the segregation spectrum spans about 250 kJ/mol; as a fraction of the total spread of the segregation spectrum, the MAE is uniformly below about 5%. To test the extrapolability of the high-fidelity framework, we report the mean (and standard deviation) absolute errors using 5-fold cross-validation in Supplementary Table 1, which shows that the fitted models are able to generalize well to the unseen folds of the dataset (with similar errors as reported in Fig. 2 for the 50/50 holdout method, and low standard deviation across the folds). We note that although most of the surveyed base-metals have fcc lattice structure, the ML framework seems to be insensitive to the lattice structure, as it similarly performs well for bcc (Fe), and hcp (Zr) metals. Therefore, we conclude that the high-fidelity ML model can be used to accurately model GB segregation across the GB and alloy spaces.

### Accelerated ML model for GB segregation

**Fig. 3:**
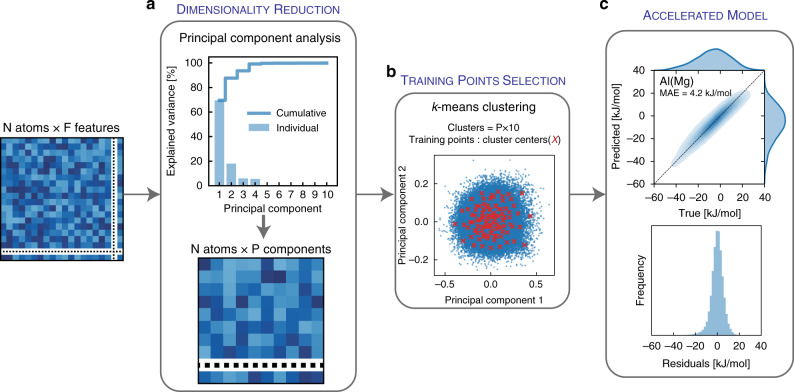
Accelerated ML model for GB segregation. For the Al(Mg) alloy, shown in Fig. 1, (**a**) principal component analysis is used to reduce the dimensionality of the feature matrix by projecting the F^SOAP^ features into P = 10 components that capture >99% of the variance. **b** *k*-means clustering is then used to divide the 10-d transformed feature space into P × 10 = 100 similar clusters; the closest GB sites to the cluster centers (shortest Euclidean distance) are used as training data-points for the learning algorithm (linear regression). **c** Predictive performance—mean absolute error (MAE)—of the accelerated model across the full Al GB network.

In alloy design, it is of interest to be able to quickly scan across the alloy space for interesting combinations. In the context of GB segregation, for example, significant efforts have been conducted to screen for nanocrystalline stabilizing elemental combinations^15,43^, complexion forming combinations^44^, or GB embrittling solute additions^45–47^. Though the high-fidelity ML model is highly accurate, it still requires ~10^4^ data points for training and fitting its ~10^3^ coefficients (features). To reduce the training cost and permit a broader scan across the full alloy space, it is appropriate to reduce the dimensions of the input features. We propose the use of unsupervised dimensionality reduction algorithms, which map a high dimensional feature vector into a low-dimensional embedding that captures its main characteristics; “unsupervised” signifies that such mapping is done without a priori knowledge the of the target value (segregation energies). As an illustration, we adopt the simplest of these algorithms, namely principal component analysis^48^, which we use to transform the F^SOAP^ = 1015 into 10 principal components (P^SOAP^) that maximize the captured variance of the feature space. We can think of this process as compressing the 1,015 features into 10 components; such compression captures >99% of the variance of the SOAP feature matrix of the Al polycrystal, as shown in Fig. 3.

For an accelerated option of the ML framework, we propose using the 10 principal components obtained from PCA as the input for the linear regression algorithm^49,50^. As the problem is now reduced to fitting P^SOAP^ + 1 coefficients (instead of F^SOAP^ + 1), we conservatively only need ~P × 10 = 100 data-points for training; 100 molecular statics computations involving the substitution of a single solute atom at a grain boundary site in a polycrystal give insight on the entire segregation spectrum. As for the selection of the 100 training data points, though random selection can be used, this could be a biased approach due to the low number of points accessing only a prevalent subset of the GB feature space in a given polycrystalline structure. Instead, we propose using *k*-means clustering^51,52^ to partition the reduced feature space into *k*=100 clusters that minimize within-cluster variances. We then use the cluster centroids to identify optimal training data-points (i.e., shortest Euclidean distance to the centroids), as shown in Fig. 3, for which GB segregation is computed, and use it to train the accelerated model. Such an approach is computationally inexpensive and ensures the full coverage of the feature space in our training dataset.

Similar to the high-fidelity model, the accelerated one can be fully automated and requires minimal input from the user. To compare the performance of both approaches, an accelerated model for Al(Mg), trained with only 100 GB sites, results in an MAE of 4.2 kJ/mol for predictions of the full ~10^5^ GB sites, compared to an MAE of 2.5 kJ/mol from the high-fidelity model trained with 50% of GB sites. This reduction (two orders of magnitude) in the required training data points, with minimal loss of accuracy, is significant, and showcases the power of the accelerated model to quickly, and accurately, predict the segregation spectra in binary alloys. It also signifies that the full GB space could possibly be reduced to a small number of key GB environments—also known as GB “building blocks”^31^—that decipher the features of the full space. We expect this to be a significant direction of future work in the context of grain boundary segregation.

Using the accelerated approach, we build ML models to predict solute segregation spectra in polycrystals for every aluminum, and magnesium, and transition metal-based binary alloys (Supplementary Figs. 2–20) that have interatomic potentials in the NIST Interatomic Potentials Repository—a total of 259 alloys (see Supplementary Fig. 1). This segregation database not only allows us to screen the alloy space for segregation “hot-spots” or regions of interest, but also to compare the variation of the spectrum with different interatomic potentials (for alloys where more than one potential exists). To illustrate the utility of the database, we plot in Fig. 4 all solute segregation spectra in a nickel-based alloy; Ni(Ag)^37^ is predicted to be highly segregating, and the opposite for Ni(Al)^53^.

**Fig. 4:**
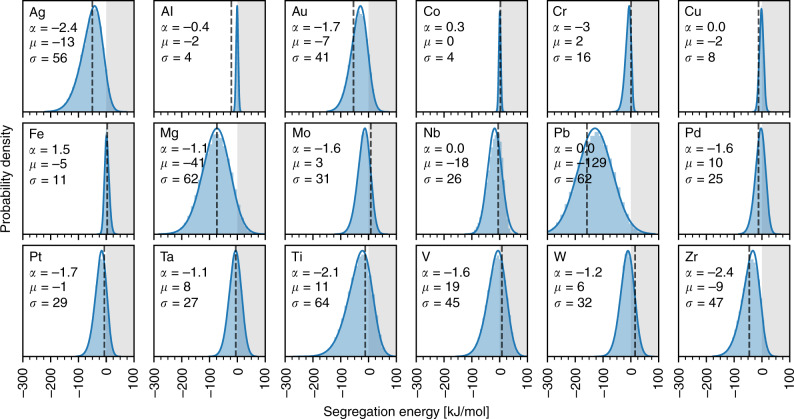
GB segregation spectra in Ni-based alloys. Using the accelerated ML model outlined in Fig. 3, we compute GB segregation energy spectra for 18 solutes^37,40,42,53,58,75–81^ in a 20x20x20 nm^3^ Ni polycrystal of grain size 10 nm; the anti-segregation region (${\Delta}E_i^{{\rm{seg}}} > 0$) is shaded. The spectra are fitted to the skew-normal function Eq. (3) (solid line), and the value of the characteristic energy *μ* (kJ/mol), width *σ* (kJ/mol), and shape *α* of the function are listed. Also, the spectra are compared to the “average” segregation energy (dashed vertical line) used to charecterize these alloys in the database of Murdoch and Schuh^55^.

## Discussion

There are three key findings to the spectral segregation database (Fig. 4 and Supplementary Figs. 2–20). The first is that all segregation spectra in all binary alloys surveyed, as hypothesized earlier in our study of the Al(Mg) system^20^, are captured well by a skew-normal function (the fitted probability density function has an R^2^ > 0.95 in all but one alloy with an R^2^=0.80; see Supplementary Figs. 2–20). This function involves three parameters—the characteristic energy *μ*, width σ and shape *α* of the distribution:

1 $$F_i^{{\rm{gb}}}({\Delta}E_i^{{\rm{seg}}}) = \frac{1}{{\sqrt {2\pi } \,\sigma }}\exp \left[ { - \frac{{\left( {{\Delta}E_i^{{\rm{seg}}} - \mu } \right)^2}}{{2\sigma ^2}}} \right]{\mathrm{erfc}}\left[ { - \frac{{\alpha \left( {{\Delta}E_i^{{\rm{seg}}} - \mu } \right)}}{{\sqrt 2 \,\sigma }}} \right]$$

These parameters are provided in the corresponding figure for each alloy considered. The second key finding is that using a McLean “average”^54^ segregation energy to characterize a binary alloy, which is the segregation literature norm^55,56^, misses key information about the accessible segregation states at the GB network. For example, the Ni(Ag) system that has a reported “average” segregation energy of −50 kJ/mol^55^, has approximately 15% of its GB network with segregation energies more than twice that, below −100 kJ/mol, as shown in the first panel of Fig. 4. GB segregation occurs first in the lowest energy states, and before a grain boundary in Ni(Ag) would experience the McLean average segregation energy, it would lie at an extremely high composition of approximately 50 atom% Ag. The knowledge of the full spectrum is thus essential to enable the design of microstructures^57^ that maximize the desired tail of the segregation spectrum (i.e. either promote or inhibit segregation). The third key finding is that, for alloys with more than one available interatomic potential, the computed segregation spectra can be sensitive to the choice of the potential. For example, potentials for the Al(Ni) system produce completely different segregation spectra, as shown in Supplementary Fig. 3, which range from having almost all GB sites being unfavorable to segregation^58^ to the complete opposite^59^; such variation can result in an order of magnitude difference in predictions for GB solute concentration even at low total solute concentrations in the system (see Supplementary Fig. 23). Further work is needed in the future to quantify the accuracy of such potentials for GB segregation studies^60^, and as always with atomistic models, it is important to remember that the present framework will only return physically reasonable results if the potential is specifically suitable for the problem at hand. For now, we report all of them, and leave the selection step to the judgment of the user.

Though the analysis in Figs. 1–3 shows that the ML models faithfully reproduce most of the details of the GB segregation spectrum, this is not the most critical test for their practical viability; these models are only useful to the extent that they correctly capture GB segregation in some realistic situation. Thus, the most important metric is the prediction for the equilibrium GB segregation state (i.e. extent of segregation). For a spectrum of segregation energies at the GB network, the equilibrium solute distribution among the different sites follows Fermi-Dirac statistics^18–20^. In a closed system with finite grain sizes, the total solute concentration X^tot^ is fixed and shared by the bulk (intra-grain) and GB solute concentrations, *X*^*c*^ and $\bar X^{gb}$, respectively, according to the GB site fraction *f*^gb^:

2 $$X^{{\rm{tot}}} = \left( {1 - f^{{\rm{gb}}}} \right)X^c + f^{{\rm{gb}}}\bar X^{{\rm{gb}}}$$

The equilibrium *X*^c^ and $\bar X^{{\rm{gb}}}$ are a function of the temperature *T*, the distribution of GB segregation energies $F_i^{{\rm{gb}}}$(${\mathrm{{\Delta}}}E_i^{{\rm{seg}}}$), and are obtained by numerically solving for *X*^*c*^ that satisfies the expanded form of Eq. (2)^20^:

3 $$X^{{\rm{tot}}} = \left( {1 - f^{{\rm{gb}}}} \right)X^c + f^{{\rm{gb}}}\mathop {\sum}\limits_i {F_i^{{\rm{gb}}}\left[ {1 + \frac{{1 - X^{\rm{c}}}}{{X^{\rm{c}}}}\exp \left( {\frac{{{\mathrm{{\Delta}}}E_i^{{\rm{seg}}}}}{{kT}}} \right)} \right]^{ - 1}}$$

**Fig. 5:**
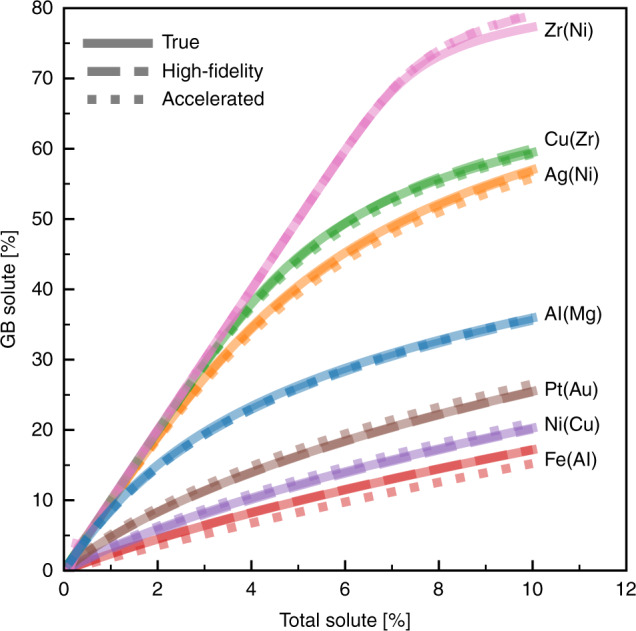
Predictions of the equilibrium segregation state. Predictions of equilibrium $\bar X^{{\rm{gb}}}$ using the true, and predicted (from both high-fidelity and accelerated ML models) segregation spectra for seven polycrystalline alloys with an average grain size of 15 nm at T= 600 K.

In Fig. 5, we compare the equilibrium GB segregation state obtained using the true computed spectrum versus the ML predicted ones with both high-fidelity and accelerated models, for all seven alloys from Figs. 1 and 2, in a polycrystal of average grain size 15 nm ($f^{{\rm{gb}}} \approx 10\%$) at T = 600 K. The predictions of the ML models closely match those of the true spectrum, indicating that the ML models capture the necessary information to correctly predict the equilibrium segregation state. Also, as briefly discussed earlier, though the value of the MAE differs from one system to another, a higher MAE does not necessarily translate to a worse result, when one normalizes to the scale of the segregation energy distribution, e.g. the Zr(Ni) system in Fig. 5. Finally, we note that the difference (deviation) in predictions of the equilibrium segregation state could be even less of an issue if the skew-normal approximation Eq. (1) is used, instead of the full discrete spectra, to quantify GB segregation using the continuous form of the segregation isotherm Eq. (3)^20^:

4 $$X^{{\rm{tot}}} = \left( {1 - f^{{\rm{gb}}}} \right)X^c + f^{{\rm{gb}}}{\int}_{ - \infty }^\infty {F_i^{{\rm{gb}}}\left[ {1 + \frac{{1 - X^{\rm{c}}}}{{X^c}}\exp \left( {\frac{{{\Delta}E_i^{{\rm{seg}}}}}{{kT}}} \right)} \right]^{ - 1}d{\Delta}E_i^{{\rm{seg}}}}$$

as the three fitted parameters (*μ*, σ, and *α*) of the skew-normal function for the true and ML predicted spectra should closely match, even for systems with high MAE, as the residuals are well-behaved and normally distributed (with a zero mean, as shown in Figs. 1 and 3).

**Fig. 6:**
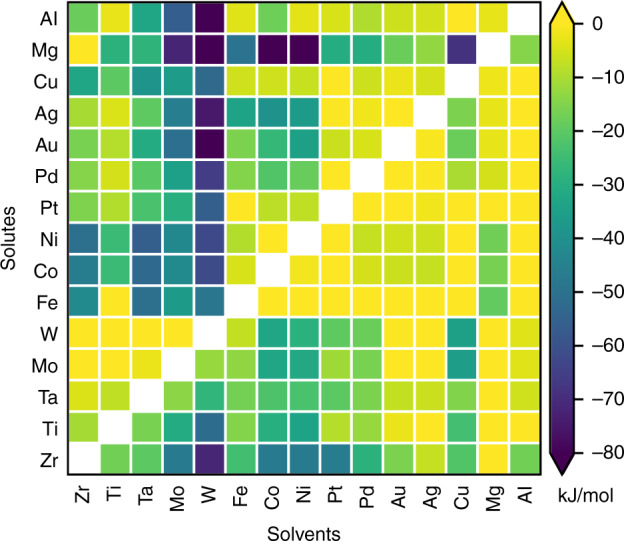
Visual summary of the predicted segregation tendency across the alloy space. The value of the first quartile of the segregation spectra (i.e. 25% of GB sites have lower segregation energies) as predicted using the accelerated model for 210 alloys (we removed columns with empty entries for compact viewing); the elements are arranged by their order on the Pettifor chemical scale^61^. For alloys with multiple interatomic potentials (see Supplementary Fig. 1), we report the least segregating spectra as a conservative choice (see Supplementary Fig. 25 for an alternative version of the figure with the most segregating spectra).

To motivate further analysis of the spectral segregation database, and visually summarize the segregation tendency across the alloy space, we plot a two-dimensional Pettifor^61^ map in Fig. 6 (for most alloys in the database) using the 25^th^ percentile value (energy) for the segregation spectra (i.e. 25% of GB sites have lower segregation energies). As the lower tail is the most enthalpically favorable, it will disproportionately influence the segregation tendency in any given alloy, especially at low or dilute solute concentration. The choice of the Pettifor chemical scale (which preserves the Mendeleev-type features of the elements^61^) is based on its success in pattern clustering (separation) for miscibility^62^, ordering tendency^63,64^, and crystal structures of intermetallics^61^ in binary alloys. Though Fig. 6 shows some clustering, it is not enough to draw concrete conclusions on the segregation tendency across the alloy space; the same finding applies to another two routinely used parameters to characterize the chemical and physical nature of the elements—electronegativity^65^, and metallic radius^66^—(see Supplementary Figs. 24–27). It is evident that more effort is needed to formulate (or extract from ML) simple phenomenological parameters (preferably derived from atomic features e.g. Miedema-style parameters^67^) that better explain these trends. We hope that this preliminary exploration of the data will promote further work on this front.

In summary, our proposed ML framework, inspired by methods developed for fitting ML-based interatomic potentials, aims to fit a “pseudo interatomic potential” for GB segregation energies in polycrystalline alloys. The framework is designed to require minimal input from the user, and as such, is automatable. As the ML literature is constantly evolving, we look forward to new developments and tools that can further improve the framework. We offered two model options. The first is a high-fidelity model that uses a large SOAP vector (>10^3^ features), a conservative radial cutoff (6 Å), and linear regression. The second, is an accelerated model that uses PCA to transform the original features into a few (10) principal components (which are then used as input features to linear regression); this reduces the dimension of the learning problem to just 100 key GB environments, which are selected by k-means clustering to ensure coverage of the GB space. The accelerated model is used to build an extensive database for segregation spectra in 259 binary alloys, which is included the Supplementary Information. We look forward to applications of this database in alloy design, and hope it motivates more widespread use of spectral approaches to GB segregation in polycrystalline materials.

## Methods

### GB segregation enthalpies

The atomistic simulation package LAMMPS^68,69^ is used for all molecular statics and dynamics simulations; OVITO^70^ is used for visualization and identification of atomic structures.

To generate the base-metal polycrystal, we fill a 20x20x20 nm^3^ volume with 16 randomly oriented grains using Voronoi tesselations with Atomsk^71^. The polycrystal is thermally annealed at 0.3–0.5 of the melting temperature under a Nose-Hoover thermostat/barostat for 250 ps using a time step of 1 fs, which relaxes the grain structure and boundaries without permitting exaggerated grain growth; this is followed by slow cooling to 0 K at a cooling rate of 3 K/ps, and a final conjugate gradient energy minimization.

To compute the spectrum of segregation enthalpies in a binary alloy, we follow the procedure in ref. ^20^. We first relax the base-metal polycrystal using the interatomic potential of that alloy, by applying an external pressure of zero in a conjugate gradient minimization, followed by a second conjugate gradient minimization (with no applied pressure). This is necessary to scale the cell, and correct for minor differences in the equilibrium lattice parameter of the base-metal across the different interatomic potentials (for example, the Ni polycrystal is thermally annealed using an interatomic potential^42^ that is fitted to Ni lattice parameter of 3.518 Å, but the Ni(Al)^53^ is fitted to 3.520 Å). Then, every GB site in the annealed polycrystal is identified using adaptive-common neighbor analysis method^72^; all atoms that have a different atomic structure than the base metal are assumed to be GB atoms. For every GB site (i), its ${\mathrm{{\Delta}}}E_i^{{\rm{seg}}}$ is calculated as the relaxed energy difference between the solute atom occupying the GB site, versus a bulk (intra-grain) site: ${\mathrm{{\Delta}}}E_i^{{\rm{seg}}} = E_{{\rm{gb}},i}^{{\rm{solute}}} - E_{\rm{c}}^{{\rm{solute}}}$; the relaxation of each state is achieved using a conjugate gradient minimization, and the reference bulk site for $E_c^{solute}$ is chosen as the center of a 6 nm sphere of the pure solvent (in the polycrystal), to avoid any long-range interactions with GB atoms. All calculations are at 0 K, isolating the enthalpic portion of the segregation energy for each site.

### Machine Learning

For feature extraction, the LAE of every GB site within a cutoff radius of 6 Å is described using the SOAP method, as implemented in the QUIP/GAP software package^28,30^. SOAP fits a set of radial basis functions and spherical harmonics to Gaussian particle density functions placed over all neighboring atoms in the LAE. The maximum number of radial basis functions (*n*_max_), degree of spherical harmonics (*l*_max_), and the width of Gaussian functions (*σ*_at_) control the size and resolution of the SOAP feature vector. We use *n*_max_ = *l*_max_ = 12 and *σ*_at_ = 1 Å for all alloys, which gives a SOAP vector with 1015 features. As for the other components of the ML framework: linear regression, principal component analysis, and *k*-means clustering are used as implemented in the Scikit-learn^73^ python package.

## Supplementary information

Supplementary Information

## Data Availability

The database for segregation spectra of all 250+ binary alloys, in the form of LAMMPS text dump files of solvent polycrystals with predicted GB solute segregation energies, is available at 10.5281/zenodo.4107058. Additional data related to this work are available from the authors upon request.

## Code availability

An example Jupyter Notebook with all necessary code to train and use the high-fidelty and accelerated machine learning models is also available at 10.5281/zenodo.4107058.

## References

[1] Lejček P, Hofmann S (1995). Thermodynamics and structural aspects of grain boundary segregation. Crit. Rev. Solid State Mater. Sci..

[2] Seah MP (1980). Chemistry of solid–solid interfaces — A review of its characterization, theory, and relevance to materials science. J. Vac. Sci. Technol..

[3] Liu CT, White CL, Horton JA (1985). Effect of boron on grain-boundaries in Ni3Al†. Acta Met..

[4] Wu R, Freeman AJ, Olson GB (1994). First principles determination of the effects of phosphorus and boron on iron grain boundary cohesion. Science.

[5] Yang T (2020). Ultrahigh-strength and ductile superlattice alloys with nanoscale disordered interfaces. Science.

[6] Rogers HC (1968). Hydrogen Embrittlement of Metals. Science.

[7] Schweinfest R, Paxton AT, Finnis MW (2004). Bismuth embrittlement of copper is an atomic size effect. Nature.

[8] Briant CL, Andresen PL (1988). Grain boundary segregation in austenitic stainless steels and its effect on intergranular corrosion and stress corrosion cracking. Metall. Trans. A.

[9] Duarte MJ (2013). Element-resolved corrosion analysis of stainless-type glass-forming steels. Science.

[10] Harmer MP (2011). The phase behavior of interfaces. Science.

[11] Frolov T, Olmsted DL, Asta M, Mishin Y (2013). Structural phase transformations in metallic grain boundaries. Nat. Commun..

[12] Raabe D (2014). Grain boundary segregation engineering in metallic alloys: A pathway to the design of interfaces. Curr. Opin. Solid State Mater. Sci..

[13] Kirchheim R (2002). Grain coarsening inhibited by solute segregation. Acta Mater..

[14] Koch CC, Scattergood RO, Darling KA, Semones JE (2008). Stabilization of nanocrystalline grain sizes by solute additions. J. Mater. Sci..

[15] Chookajorn T, Murdoch HA, Schuh CA (2012). Design of stable nanocrystalline alloys. Science.

[16] Herbig M (2013). Atomic-scale quantification of grain boundary segregation in nanocrystalline material. Phys. Rev. Lett..

[17] Steigerwald DA, Wynblatt P (1988). Calculation of the anisotropy of equilibrium surface composition in metallic solid solutions using the embedded atom method. Surf. Sci..

[18] White CL, Stein DF (1978). Sulfur segregation to grain boundaries in Ni3Al and Ni3(AI,Ti) alloys. Metall. Trans. A.

[19] Kirchheim R (1988). Hydrogen solubility and diffusivity in defective and amorphous metals. Prog. Mater. Sci..

[20] Wagih M, Schuh CA (2019). Spectrum of grain boundary segregation energies in a polycrystal. Acta Mater..

[21] Becker CA, Tavazza F, Trautt ZT, Buarque De Macedo RA (2013). Considerations for choosing and using force fields and interatomic potentials in materials science and engineering. Curr. Opin. Solid State Mater. Sci..

[22] Hale LM, Trautt ZT, Becker CA (2018). Evaluating variability with atomistic simulations: the effect of potential and calculation methodology on the modeling of lattice and elastic constants. Model. Simul. Mater. Sci. Eng..

[23] Huber L, Hadian R, Grabowski B, Neugebauer J (2018). A machine learning approach to model solute grain boundary segregation. npj Comput. Mater..

[24] Huber L, Grabowski B, Militzer M, Neugebauer J, Rottler J (2017). Ab initio modelling of solute segregation energies to a general grain boundary. Acta Mater..

[25] Mueller T, Hernandez A, Wang C (2020). Machine learning for interatomic potential models. J. Chem. Phys..

[26] Behler J (2016). Perspective: Machine learning potentials for atomistic simulations. J. Chem. Phys..

[27] Behler J, Parrinello M (2007). Generalized neural-network representation of high-dimensional potential-energy surfaces. Phys. Rev. Lett..

[28] Bartók AP, Payne MC, Kondor R, Csányi G (2010). Gaussian Approximation Potentials: The Accuracy of Quantum Mechanics, without the Electrons. Phys. Rev. Lett..

[29] Thompson AP, Swiler LP, Trott CR, Foiles SM, Tucker GJ (2015). Spectral neighbor analysis method for automated generation of quantum-accurate interatomic potentials. J. Comput. Phys..

[30] Bartók AP, Kondor R, Csányi G (2013). On representing chemical environments. Phys. Rev. B.

[31] Rosenbrock CW, Homer ER, Csányi G, Hart GLW (2017). Discovering the building blocks of atomic systems using machine learning: application to grain boundaries. npj Comput. Mater..

[32] Deringer VL, Caro MA, Csányi G (2019). Machine Learning Interatomic Potentials as Emerging Tools for Materials Science. Adv. Mater..

[33] Harrell, F. E. Jr *Regression Modeling Strategies: With Applications to Linear Models, Logistic and Ordinal Regression, and Survival Analysis* (Springer, 2015).

[34] Cochran, W. *Sampling Techniques* (John Wiley, 2006).

[35] Rhodes NR, Tschopp MA, Solanki KN (2013). Quantifying the energetics and length scales of carbon segregation to *α* -Fe symmetric tilt grain boundaries using atomistic simulations. Model. Simul. Mater. Sci. Eng..

[36] Mendelev MI, Asta M, Rahman MJ, Hoyt JJ (2009). Development of interatomic potentials appropriate for simulation of solid–liquid interface properties in Al–Mg alloys. Philos. Mag..

[37] Pan Z, Borovikov V, Mendelev MI, Sansoz F (2018). Development of a semi-empirical potential for simulation of Ni solute segregation into grain boundaries in Ag. Model. Simul. Mater. Sci. Eng..

[38] Borovikov V, Mendelev MI, King AH (2016). Effects of stable and unstable stacking fault energy on dislocation nucleation in nano-crystalline metals. Model. Simul. Mater. Sci. Eng..

[39] Mendelev MI, Srolovitz DJ, Ackland GJ, Han S (2005). Effect of Fe segregation on the migration of a non-symmetric ∑5 tilt grain boundary in Al. J. Mater. Res..

[40] Onat, B. & Durukanoǧlu, S. An optimized interatomic potential for Cu-Ni alloys with the embedded-atom method. *J. Phys. Condens. Matter* **26**, 035404 (2014). 10.1088/0953-8984/26/3/035404 24351396

[41] O’Brien CJ, Barr CM, Price PM, Hattar K, Foiles SM (2018). Grain boundary phase transformations in PtAu and relevance to thermal stabilization of bulk nanocrystalline metals. J. Mater. Sci..

[42] Wilson SR, Mendelev MI (2015). Anisotropy of the solid-liquid interface properties of the Ni-Zr B33 phase from molecular dynamics simulation. Philos. Mag..

[43] Darling KA (2011). Stabilized nanocrystalline iron-based alloys: Guiding efforts in alloy selection. Mater. Sci. Eng. A.

[44] Schuler JD, Rupert TJ (2017). Materials selection rules for amorphous complexion formation in binary metallic alloys. Acta Mater..

[45] Lejček P, Šob M, Paidar V (2017). Interfacial segregation and grain boundary embrittlement: An overview and critical assessment of experimental data and calculated results. Prog. Mater. Sci..

[46] Gibson MA, Schuh CA (2015). Segregation-induced changes in grain boundary cohesion and embrittlement in binary alloys. Acta Mater..

[47] Gibson MA, Schuh CA (2016). A compilation of ab-initio calculations of embrittling potencies in binary metallic alloys. Data Br..

[48] Tipping M. E., M. E. & Bishop CMBishop, C. M. *Probabilistic Principal Component Analysis* (TensorFlow, 1997).

[49] Jolliffe IT (1982). A Note on the Use of Principal Components in Regression. Appl. Stat..

[50] Helfrecht BA, Semino R, Pireddu G, Auerbach SM, Ceriotti M (2019). A new kind of atlas of zeolite building blocks. J. Chem. Phys..

[51] Lloyd, S. P. Least squares quantization in PCM. *IEEE Trans. Inf. Theory* **28**, 129–137 (1982).

[52] Elkan, C. Using the Triangle Inequality to Accelerate-Means. *ACM* 147–153 (2003).

[53] Purja Pun GP, Mishin Y (2009). Development of an interatomic potential for the Ni-Al system. Philos. Mag..

[54] McLean, D. *Grain Boundaries in Metals* (Clarendon Press, 1957).

[55] Murdoch HA, Schuh CA (2013). Estimation of grain boundary segregation enthalpy and its role in stable nanocrystalline alloy design. J. Mater. Res..

[56] Lejcek, P. *Grain Boundary Segregation in Metals* Vol. 136 (Springer, Berlin, Heidelberg, 2010).

[57] Watanabe T, Tsurekawa S (1999). Control of brittleness and development of desirable mechanical properties in polycrystalline systems by grain boundary engineering. Acta Mater..

[58] Zhou XW, Johnson RA, Wadley HNG (2004). Misfit-energy-increasing dislocations in vapor-deposited CoFe/NiFe multilayers. Phys. Rev. B - Condens. Matter Mater. Phys..

[59] Mishin Y, Mehl MJ, Papaconstantopoulos DA (2002). Embedded-atom potential for B2-NiAl. Phys. Rev. B.

[60] Hu Y, Schuler JD, Rupert TJ (2018). Identifying interatomic potentials for the accurate modeling of interfacial segregation and structural transitions. Comput. Mater. Sci..

[61] Pettifor DG (1986). The structures of binary compounds: I. phenomenological structure maps. J. Phys. C. Solid State Phys..

[62] Zhang RF (2017). An informatics guided classification of miscible and immiscible binary alloy systems. Sci. Rep..

[63] Hart GLW, Curtarolo S, Massalski TB, Levy O (2014). Comprehensive search for new phases and compounds in binary alloy systems based on platinum-group metals, using a computational first-principles approach. Phys. Rev. X.

[64] Curtarolo S (2013). The high-throughput highway to computational materials design. Nat. Mater..

[65] Allred AL (1961). Electronegativity values from thermochemical data. J. Inorg. Nucl. Chem..

[66] Teatum, E. T., Gschneidner, K. A. Jr & Waber, J. T. Compilation of Calculated Data Useful in Predicting Metallurgical Behavior of the Elements in Binary Alloy Systems, 10.2172/4789465 (1968).

[67] Miedema AR (1973). Simple model for alloys. Philips Tech. Rev..

[68] Plimpton S (1995). Fast Parallel Algorithms for Short-Range Molecular Dynamics. J. Comput. Phys..

[69] Brown WM, Kohlmeyer A, Plimpton SJ, Tharrington AN (2012). Implementing molecular dynamics on hybrid high performance computers - Particle-particle particle-mesh. Comput. Phys. Commun..

[70] Stukowski A (2010). Visualization and analysis of atomistic simulation data with OVITO–the Open Visualization Tool. Model. Simul. Mater. Sci. Eng..

[71] Hirel P (2015). Atomsk: A tool for manipulating and converting atomic data files. Comput. Phys. Commun..

[72] Stukowski A (2012). Structure identification methods for atomistic simulations of crystalline materials. Model. Simul. Mater. Sci. Eng..

[73] Pedregosa F (2011). Scikit-learn: Machine Learning in Python. J. Mach. Learn. Res..

[74] Kelchner CL, Plimpton S (1998). Dislocation nucleation and defect structure during surface indentation. Phys. Rev. B - Condens. Matter Mater. Phys..

[75] Samolyuk, G. D., Béland, L. K., Stocks, G. M. & Stoller, R. E. Electron-phonon coupling in Ni-based binary alloys with application to displacement cascade modeling. *J. Phys. Condens. Matter* **28**, 175501 (2016). 10.1088/0953-8984/28/17/175501 27033732

[76] Maisel SB, Ko WS, Zhang JL, Grabowski B, Neugebauer J (2017). Thermomechanical response of NiTi shape-memory nanoprecipitates in TiV alloys. Phys. Rev. Mater..

[77] Adams JB, Foiles SM, Wolfer WG (1989). Self-diffusion and impurity diffusion of fcc metals using the five-frequency model and the Embedded Atom Method. J. Mater. Res..

[78] Béland LK (2016). Features of primary damage by high energy displacement cascades in concentrated Ni-based alloys. J. Appl. Phys..

[79] Howells CA, Mishin Y (2018). Angular-dependent interatomic potential for the binary Ni–Cr system. Model. Simul. Mater. Sci. Eng..

[80] Zhou XW, Foster ME, Sills RB (2018). An Fe-Ni-Cr embedded atom method potential for austenitic and ferritic systems. J. Comput. Chem..

[81] Zhang Y, Ashcraft R, Mendelev MI, Wang CZ, Kelton KF (2016). Experimental and molecular dynamics simulation study of structure of liquid and amorphous Ni62Nb38 alloy. J. Chem. Phys..

