# Supplementary material for: Learning grain boundary segregation energy spectra in polycrystals
Source: Nat Commun. 2020 Dec 11;11:6376. doi: 10.1038/s41467-020-20083-6 (PMC7733488; doi:10.1038/s41467-020-20083-6)
Supplement: Supplementary file 1 — Supplementary Information [file 41467_2020_20083_MOESM1_ESM.pdf]

Supplementary Information

Learning Grain Boundary Segregation Energy Spectra in Polycrystals

Malik Wagih [1], Peter M. Larsen [2], and Christopher A. Schuh [2]

[1] Department of Nuclear Science and Engineering, Massachusetts Institute of Technology, 77 Massachusetts Avenue, Cambridge, Massachusetts 02139, USA.
[2] Department of Materials Science and Engineering, Massachusetts Institute of Technology, 77 Massachusetts Avenue, Cambridge, Massachusetts 02139, USA.

SUPPLEMENTARY NOTE 1. SPECTRAL SEGREGATION DATABASE

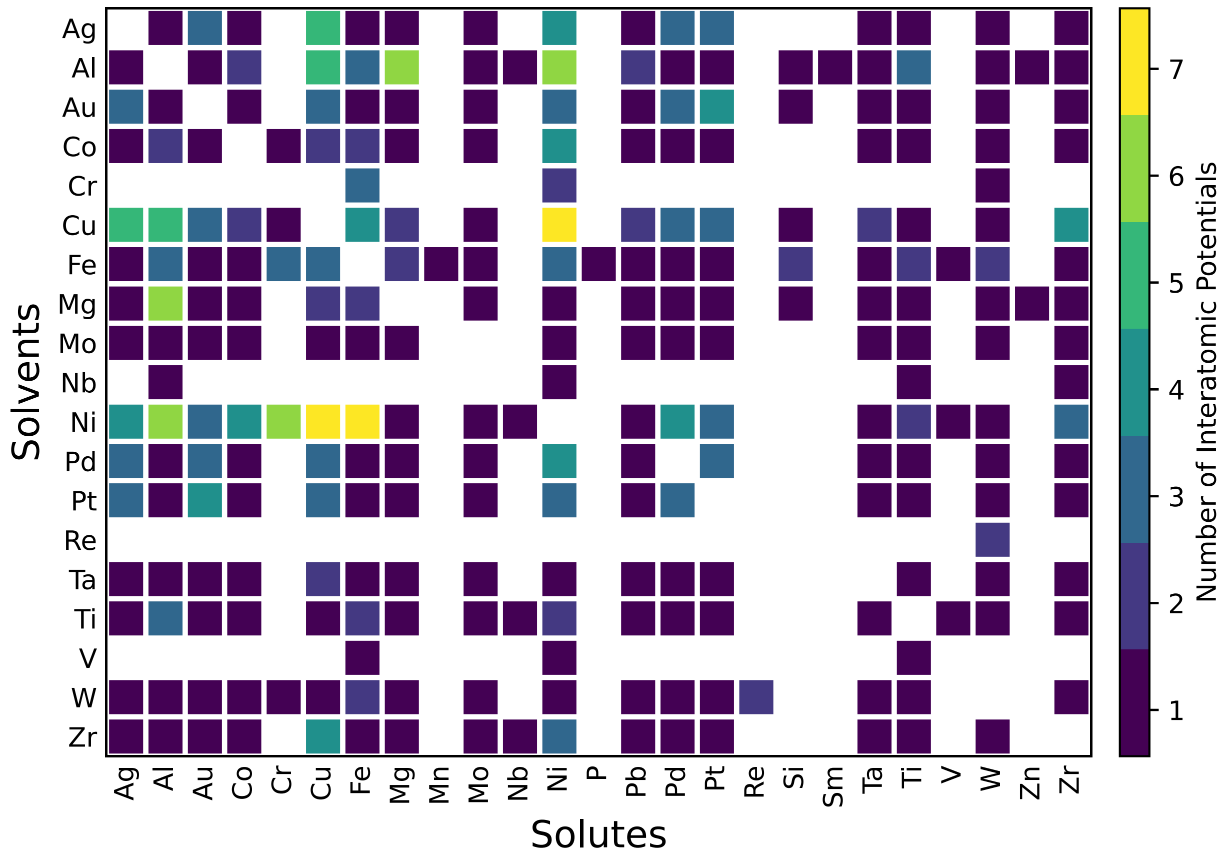

Supplementary Fig. 1 All surveyed binary alloys and their existing interatomic potentials – a total of 260 alloys and 434 combinations of interatomic potentials. We sought to report all interatomic potentials without judging their relevance to segregation studies, but we 1) limited solvents to bcc, fcc, and hcp metals, and 2) removed H, C, O, U, Xe solutes, and 3) excluded interatomic potentials that do not correctly predict the equilibrium 0 K lattice structure for the solvent, e.g. fcc Fe instead of bcc Fe.

Ag-based alloys

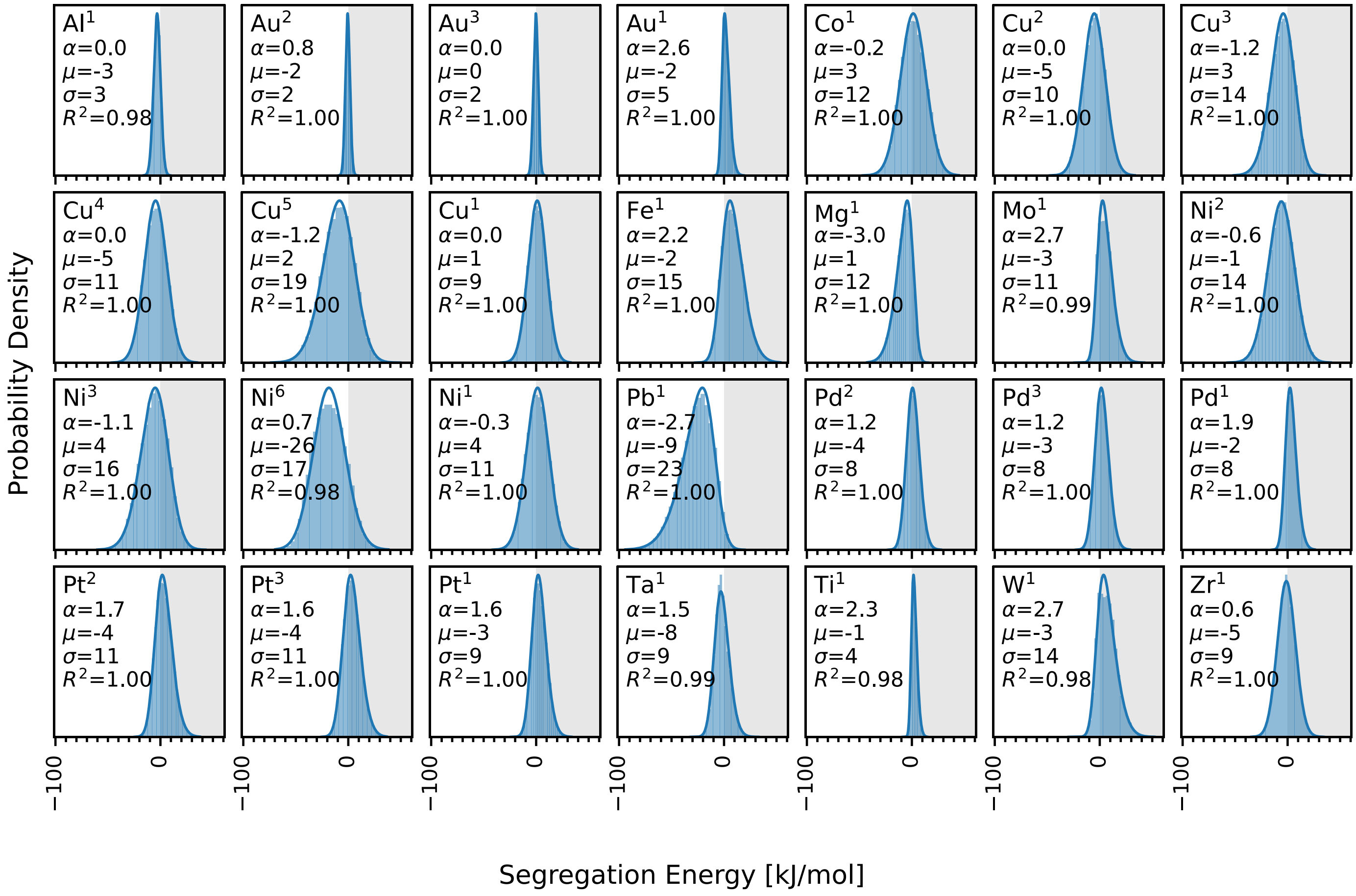

Supplementary Fig. 2 Solute segregation spectra at GBs in Ag-based alloys[1–6].

Al-based alloys

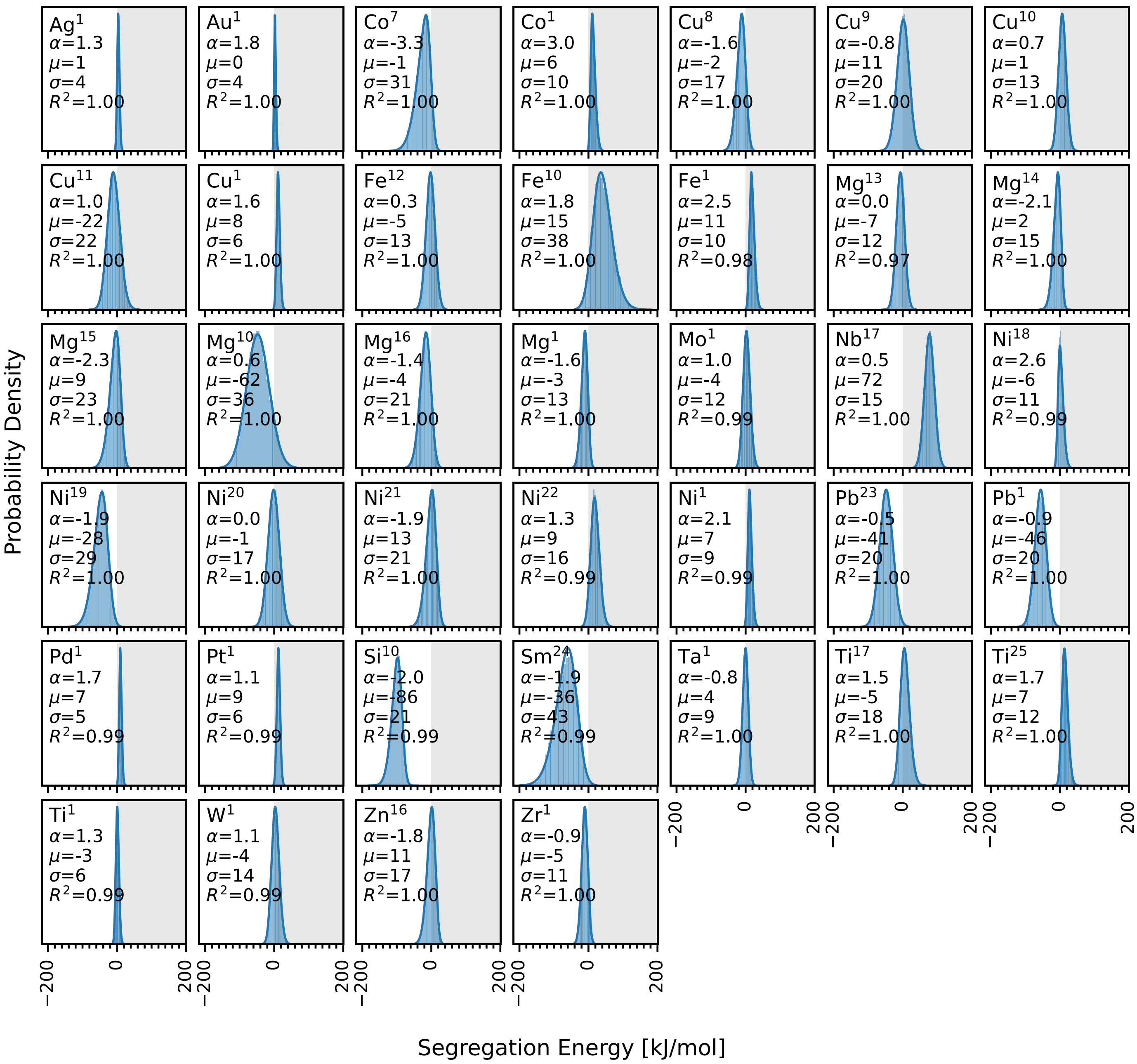

Supplementary Fig. 3 Solute segregation spectra at GBs in Al-based alloys[1,7–25].

Au-based alloys

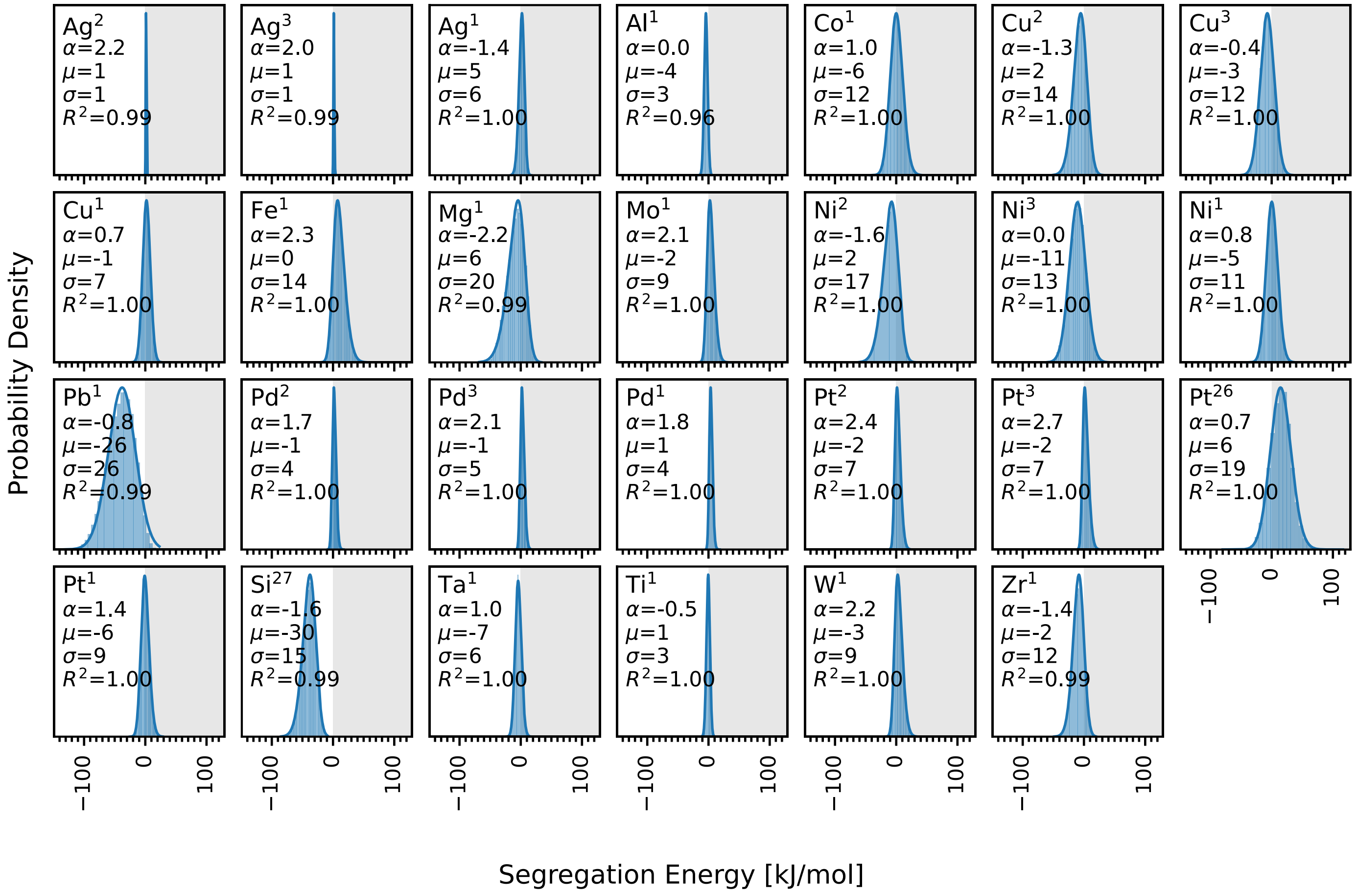

Supplementary Fig. 4 Solute segregation spectra at GBs in Au-based alloys [1–3,26,27].

Co-based alloys

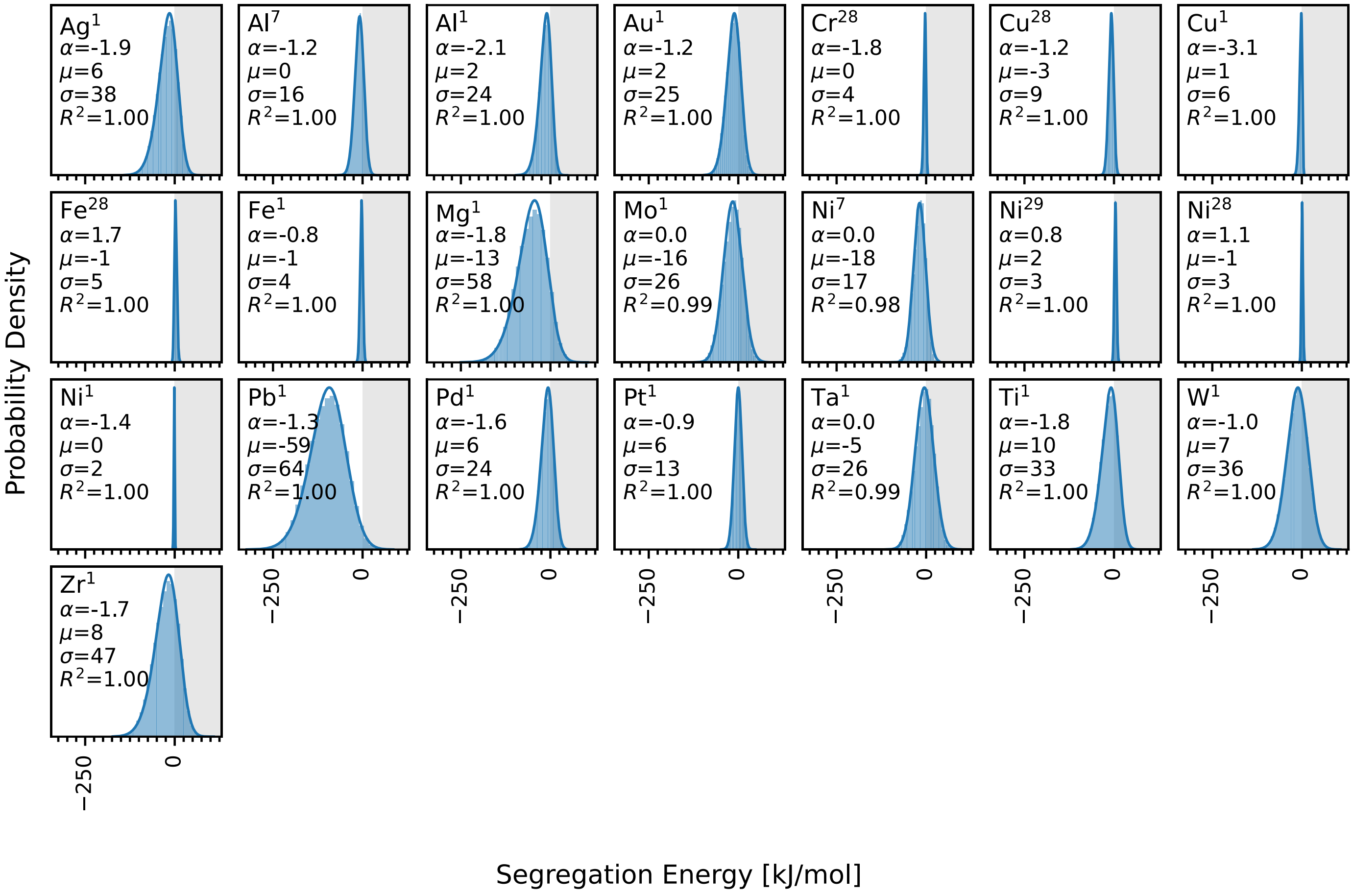

Supplementary Fig. 5 Solute segregation spectra at GBs in Co-based alloys [1,7,28,29].

Cr-based alloys

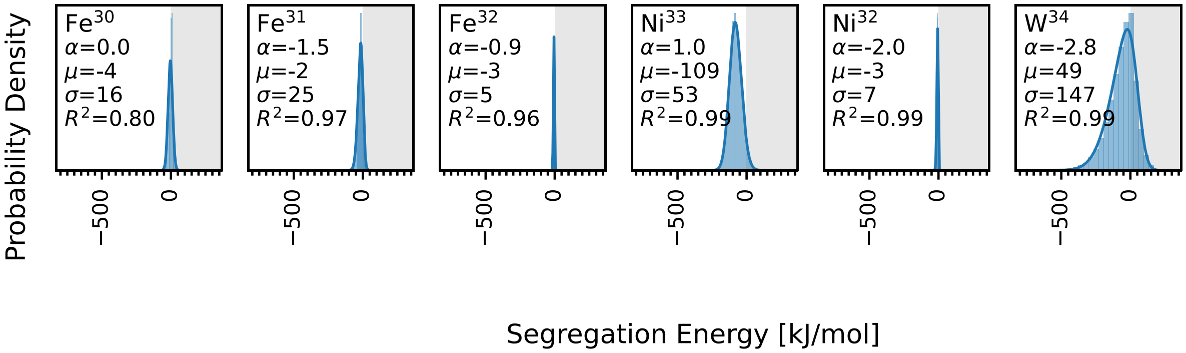

Supplementary Fig. 6 Solute segregation spectra at GBs in Cr-based alloys [30–34].

Cu-based alloys

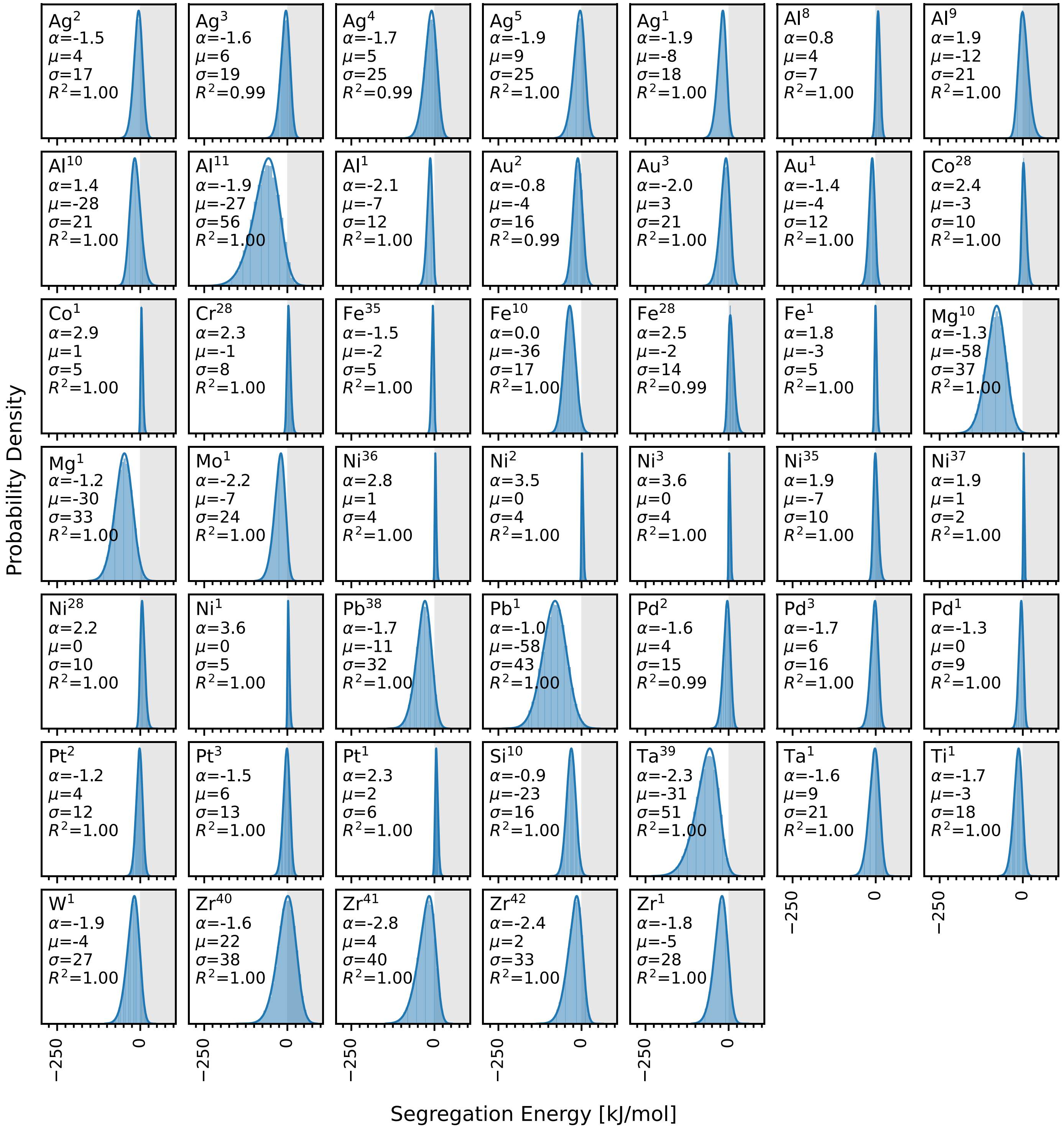

Supplementary Fig. 7 Solute segregation spectra at GBs in Cu-based alloys [1–5,8–11,28,35–42].

Fe-based alloys

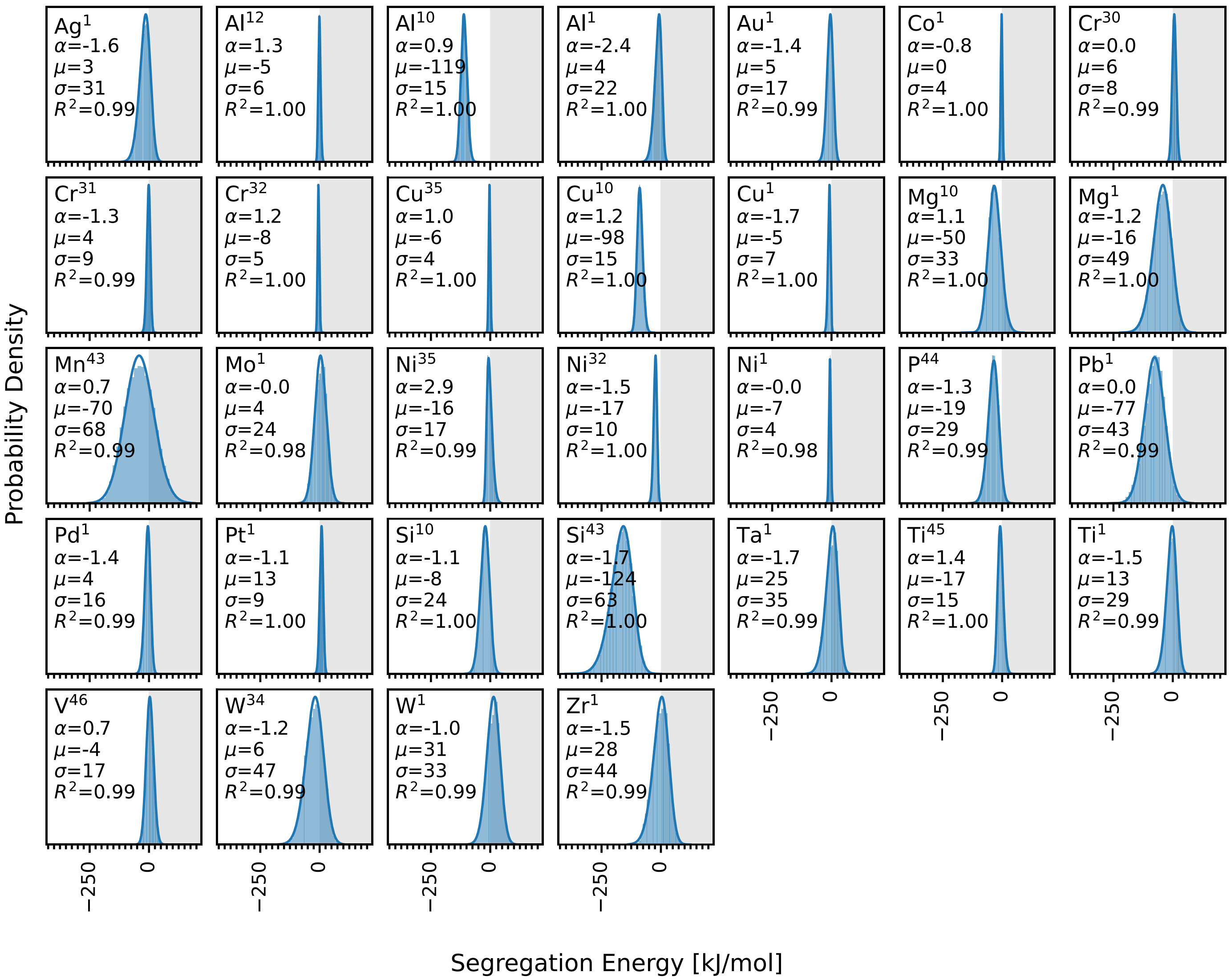

Supplementary Fig. 8 Solute segregation spectra at GBs in Fe-based alloys [1,10,12,30–32,34,35,43–46].

Mg-based alloys

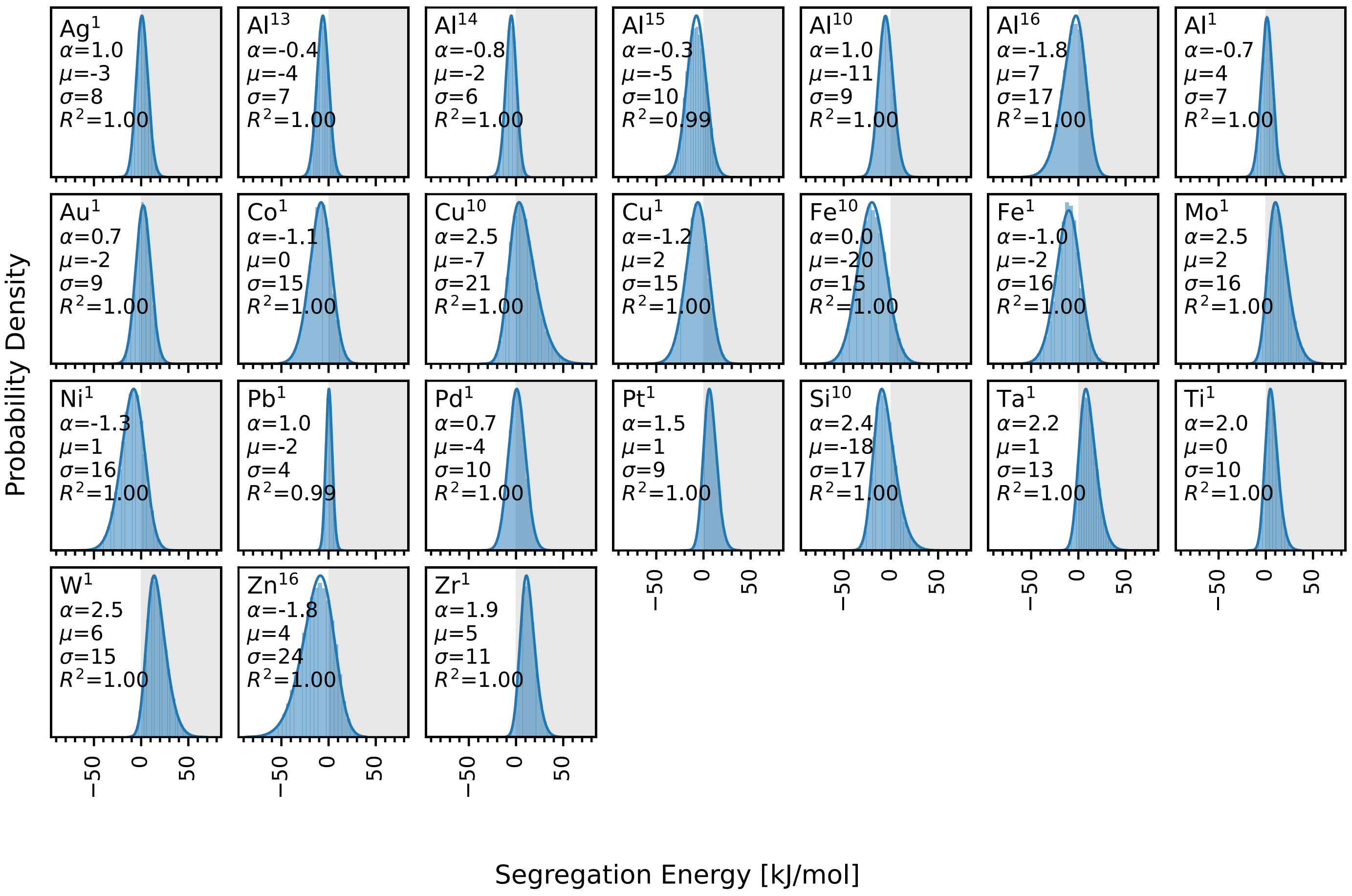

Supplementary Fig. 9 Solute segregation spectra at GBs in Mg-based alloys[1,10,13–16].

Mo-based alloys

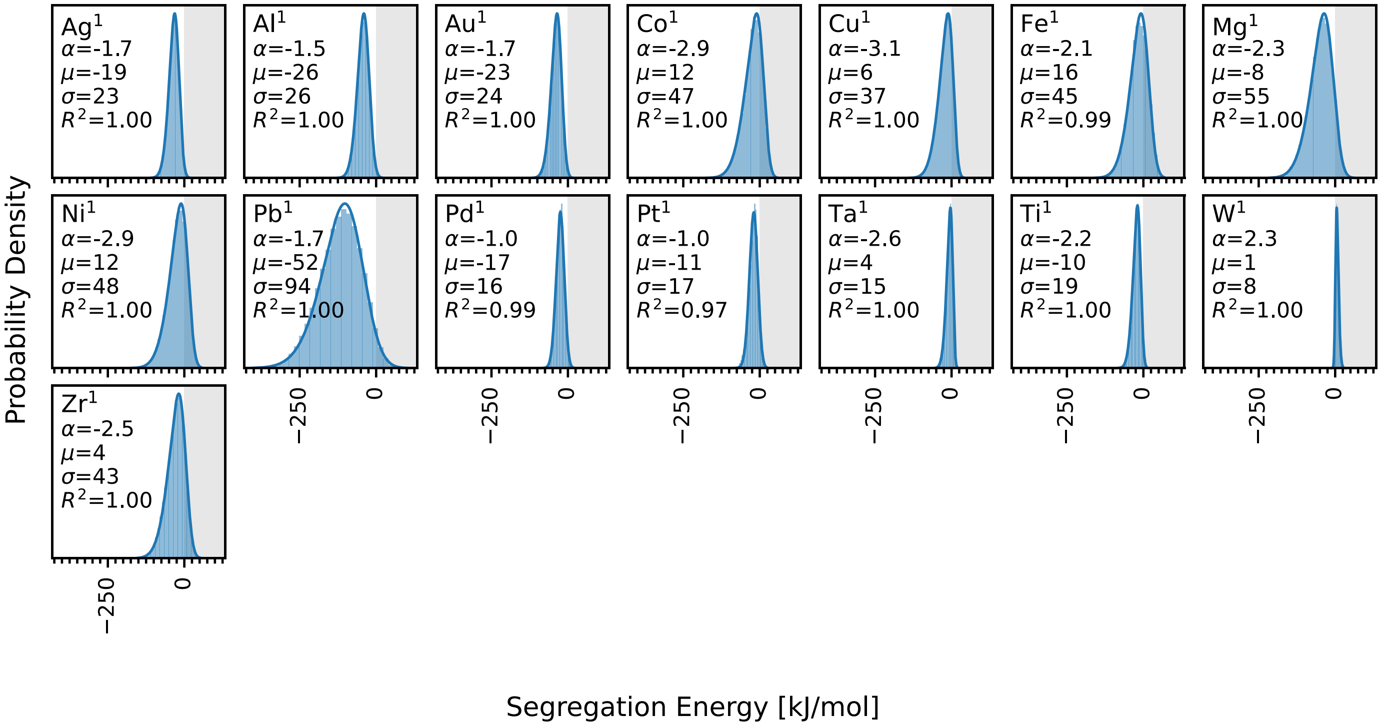

Supplementary Fig. 10 Solute segregation spectra at GBs in Mo-based alloys[1].

Nb-based alloys

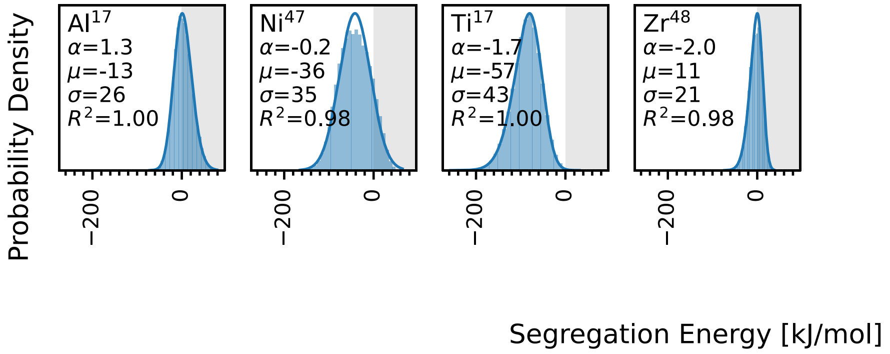

Supplementary Fig. 11 Solute segregation spectra at GBs in Nb-based alloys[17,47,48].

Ni-based alloys

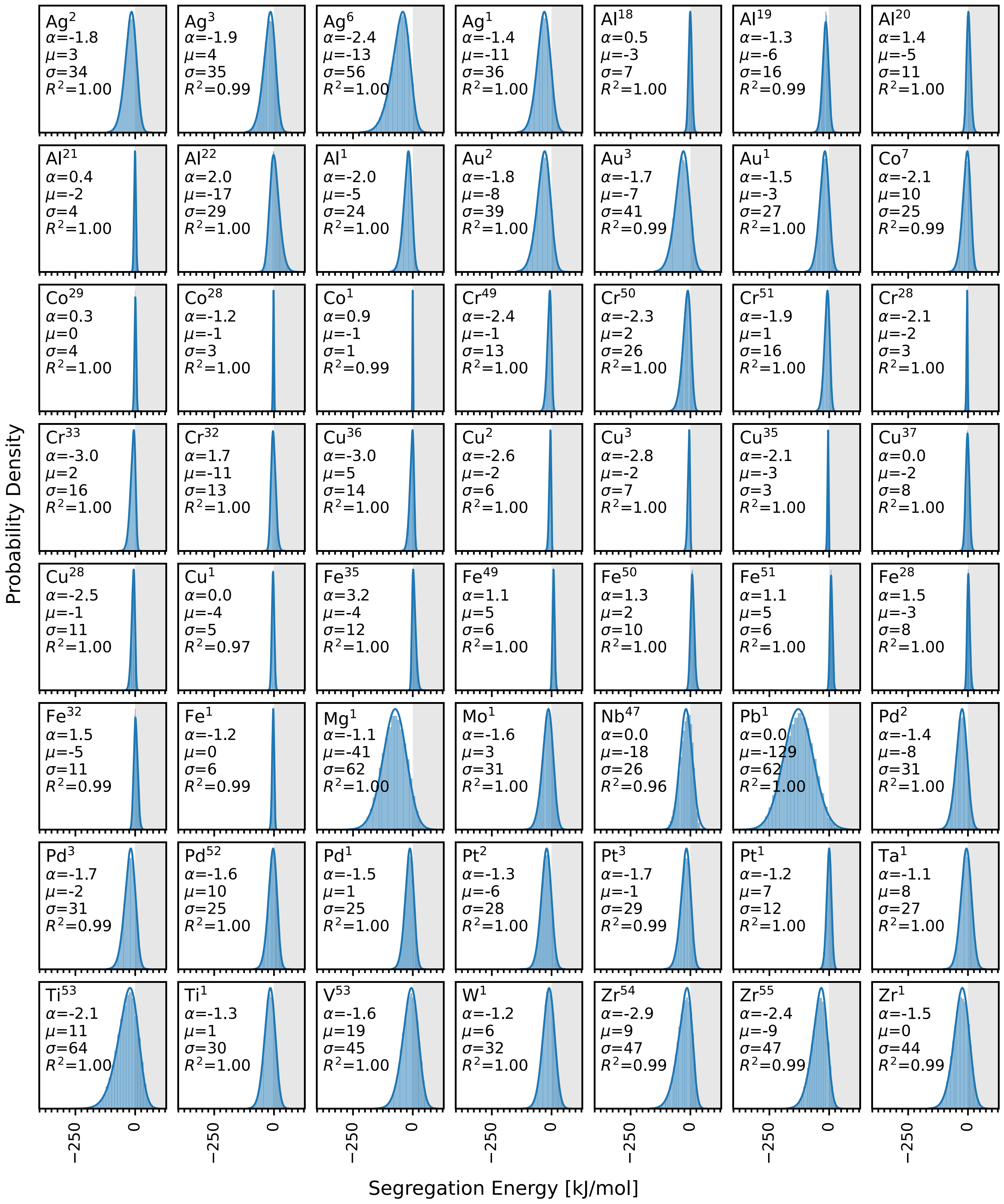

Supplementary Fig. 12 Solute segregation spectra at GBs in Ni-based alloys[1–3,6,7,18–22,28,29,32,33,35–37,47,49–55].

Pd-based alloys

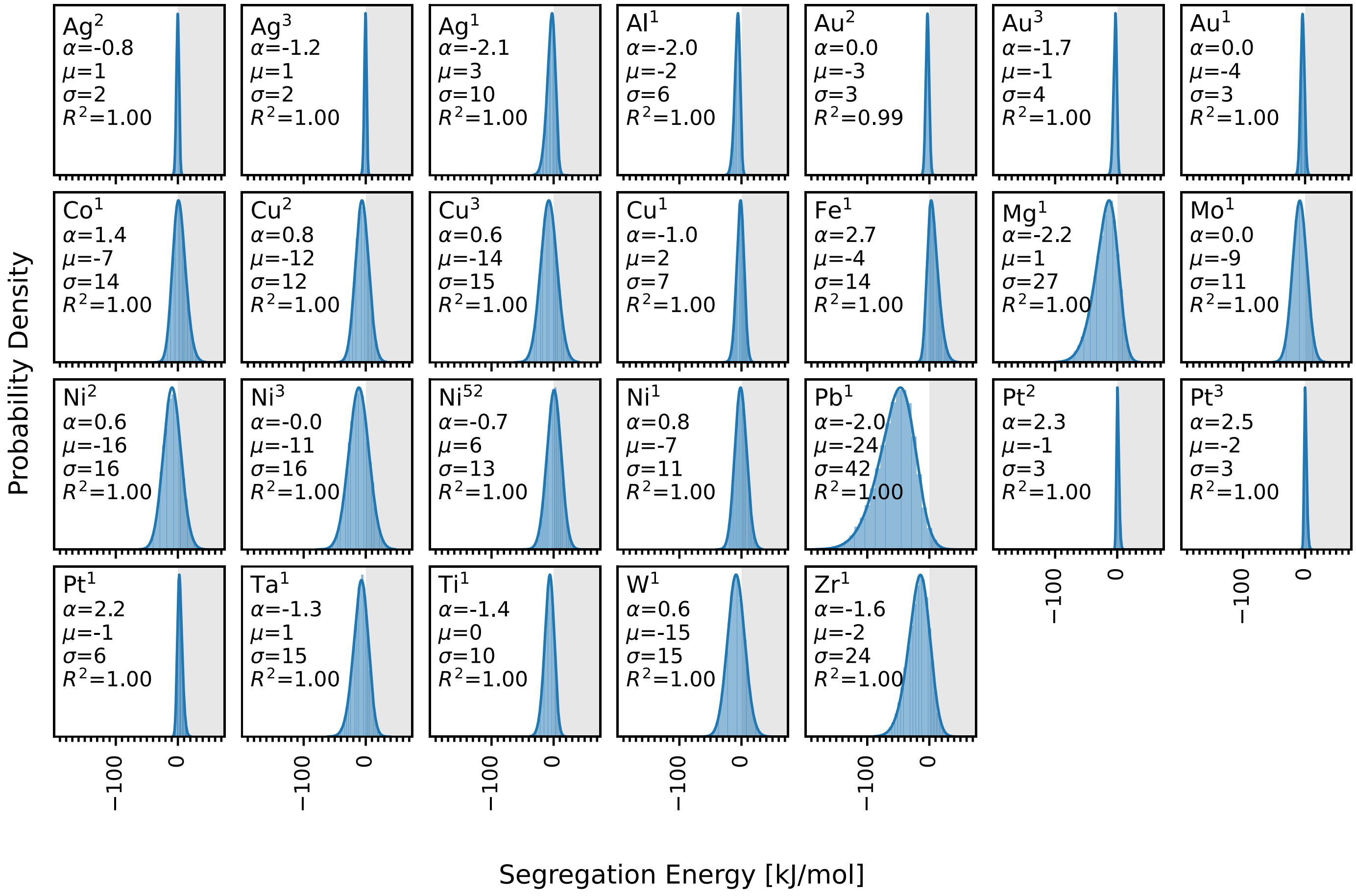

Supplementary Fig. 13 Solute segregation spectra at GBs in Pd-based alloys [1–3,52].

Pt-based alloys

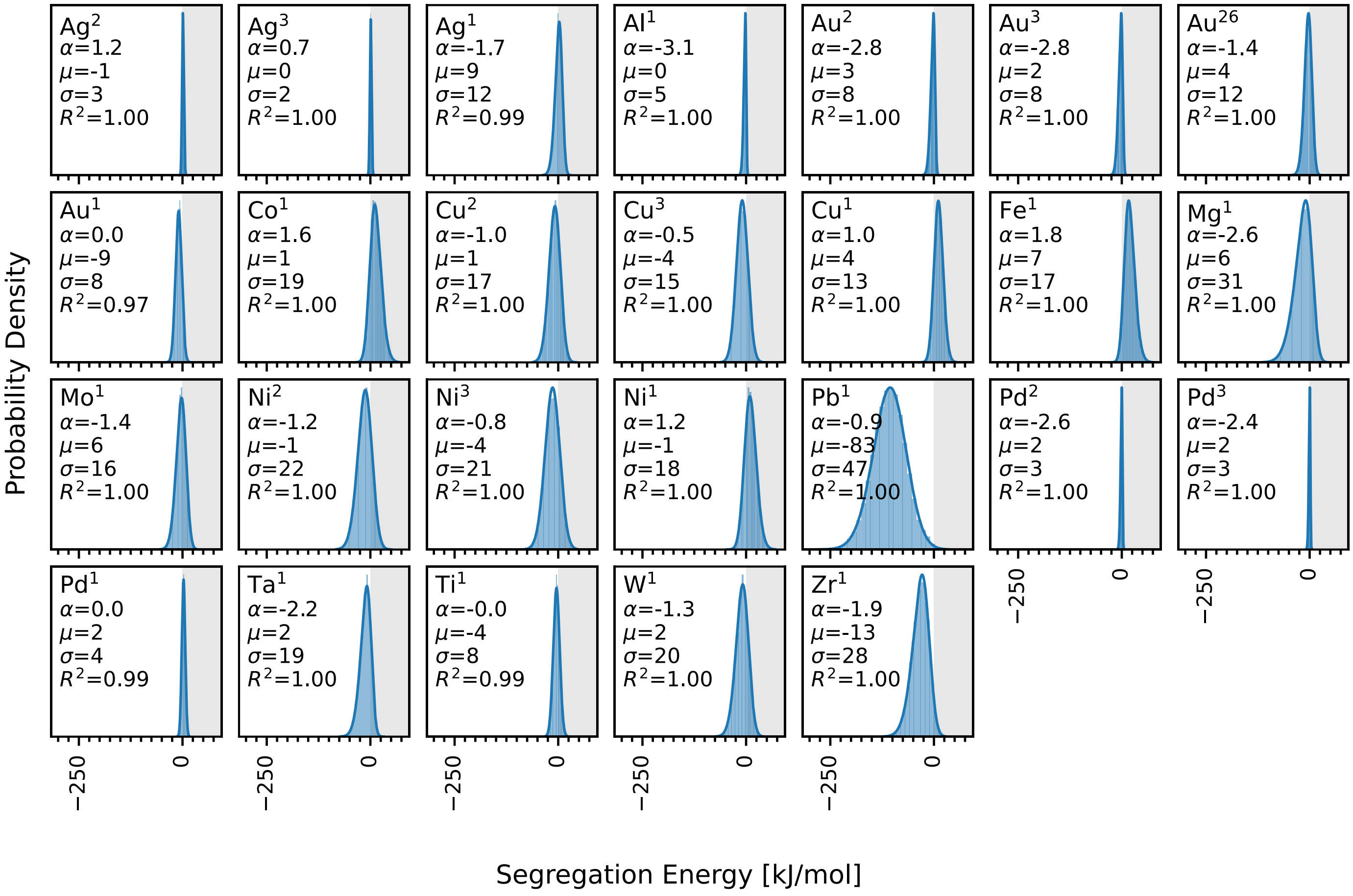

Supplementary Fig. 14 Solute segregation spectra at GBs in Pt-based alloys [1–3,26].

Re-based alloys

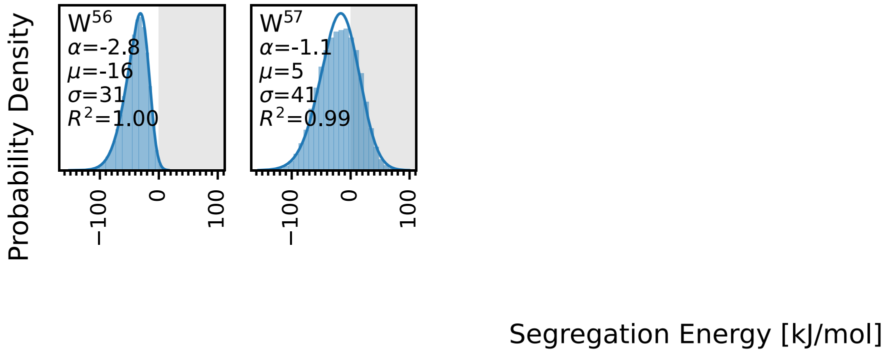

Supplementary Fig. 15 Solute segregation spectra at GBs in Re-based alloys[56,57].

Ta-based alloys

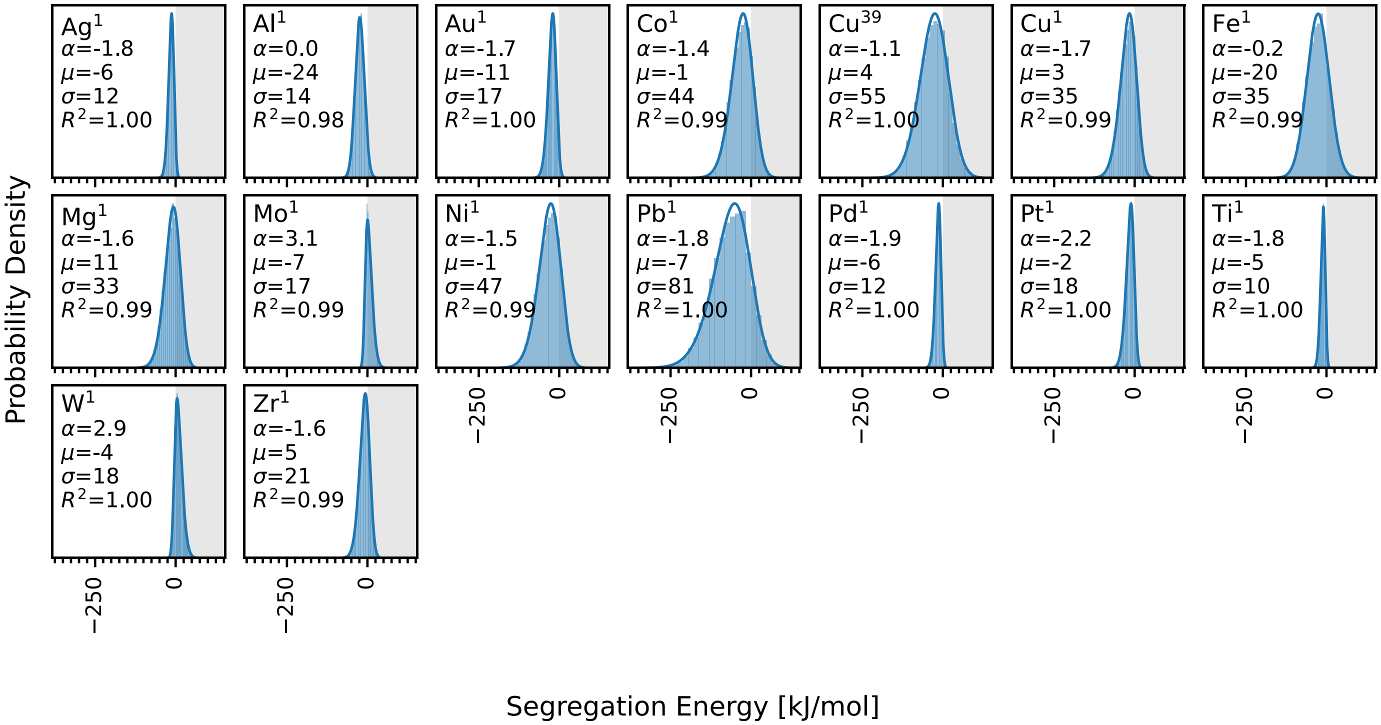

Supplementary Fig. 16 Solute segregation spectra at GBs in Ta-based alloys[1,39].

Ti-based alloys

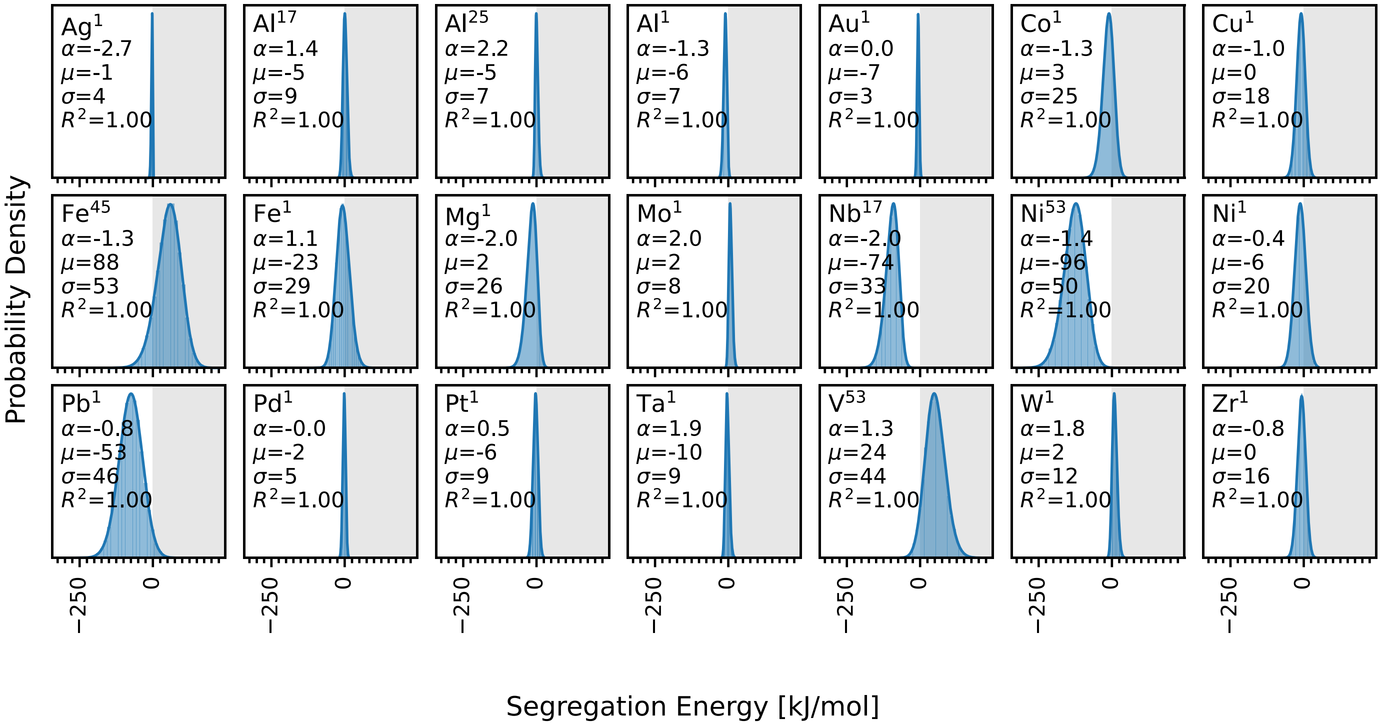

Supplementary Fig. 17 Solute segregation spectra at GBs in Ti-based alloys[1,17,25,45,53].

V-based alloys

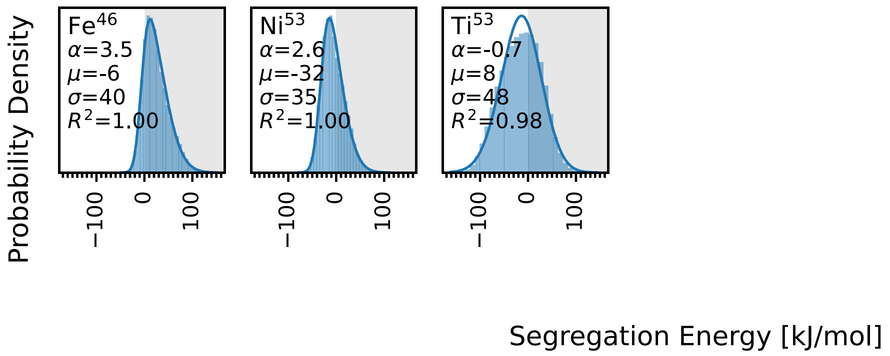

Supplementary Fig. 18 Solute segregation spectra at GBs in V-based alloys[46,53].

W-based alloys

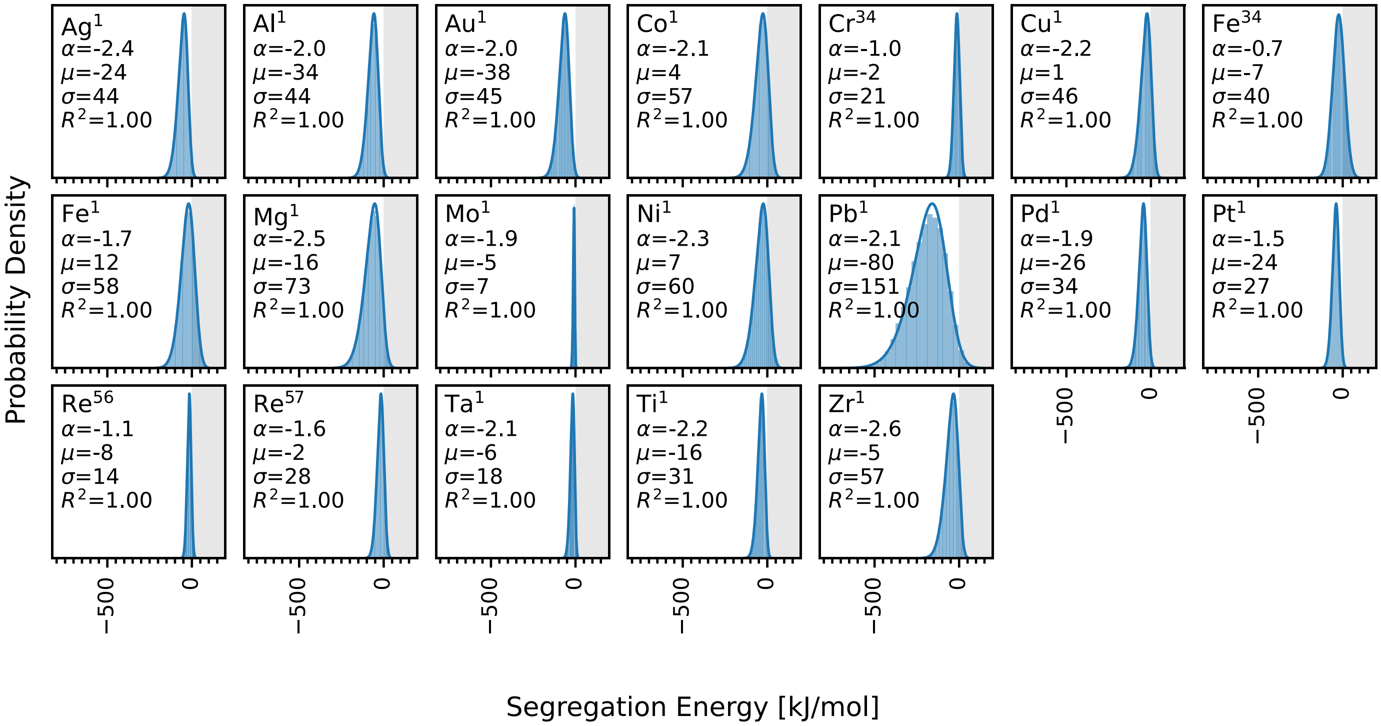

Supplementary Fig. 19 Solute segregation spectra at GBs in W-based alloys[1,34,56,57].

Zr-based alloys

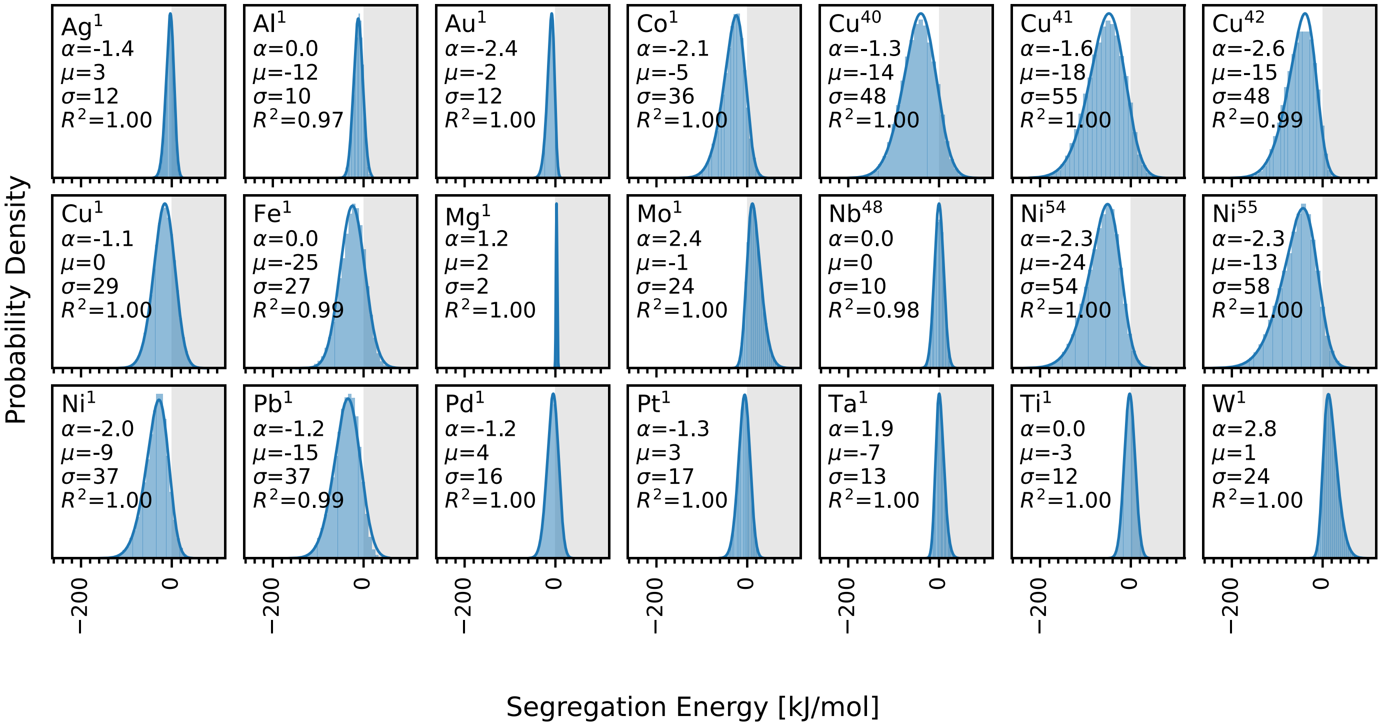

Supplementary Fig. 20 Solute segregation spectra at GBs in Zr-based alloys[1,40–42,48,54,55].

SUPPLEMENTARY NOTE 2. MACHINE LEARNING

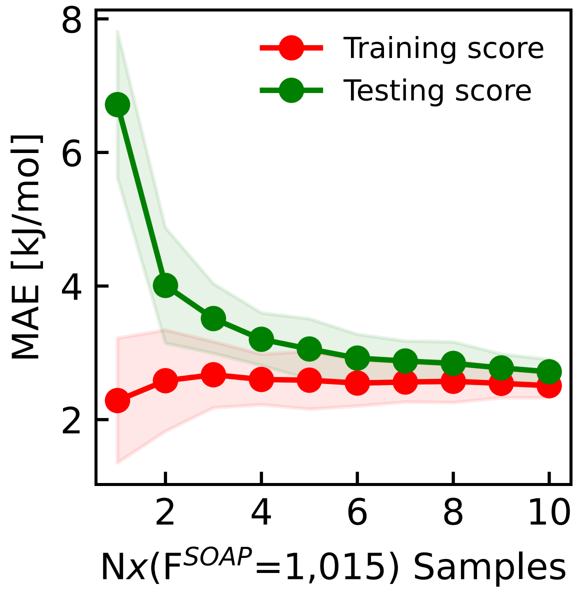

Supplementary Fig. 21 The mean absolute error of the high-fidelity model for the Al(Mg) system as a function of number of training datapoints per SOAP features (NxF^{SOAP}). The shaded region is the standard deviation for 50 repetitions of the 50/50 train/test holdout method used to train the igh-fidelity model. The plot validates the 10 points per predictor rule of thumb, as it shows the learning model to converge to a low variance and low bias model with $10xF^{SOAP}$ datapoints.

**Solvent(Solute)**	**50/50 Training/Testing** train(test) error [kJ/mol]	**5-fold Cross-Validation** mean(stdv) error [kJ/mol]
Ag(Ni)	3.38 (3.49)	3.36 (0.09)
Al(Mg)	2.38 (2.46)	2.45 (0.04)
Cu(Zr)	5.46 (5.62)	5.57 (0.10)
Fe(Al)	1.41 (1.42)	1.29 (0.07)
Ni(Cu)	0.81 (0.82)	0.86 (0.07)
Pt(Au)	0.67 (0.68)	0.69 (0.02)
Zr(Ni)	12.62 (12.96)	12.86 (0.15)

Supplementary Table 1 Predictive performance of the high-fidelity model using a) 50/50 training/testing split and b) 5-fold cross validation. The reported errors for the two methods are comparable, which indicates that a simple 50/50 split is sufficient for the model. Also, the low standard deviation (stdv) of error across the 5 folds shows the ability of high-fidelity model to extrapolate well across the GB population.

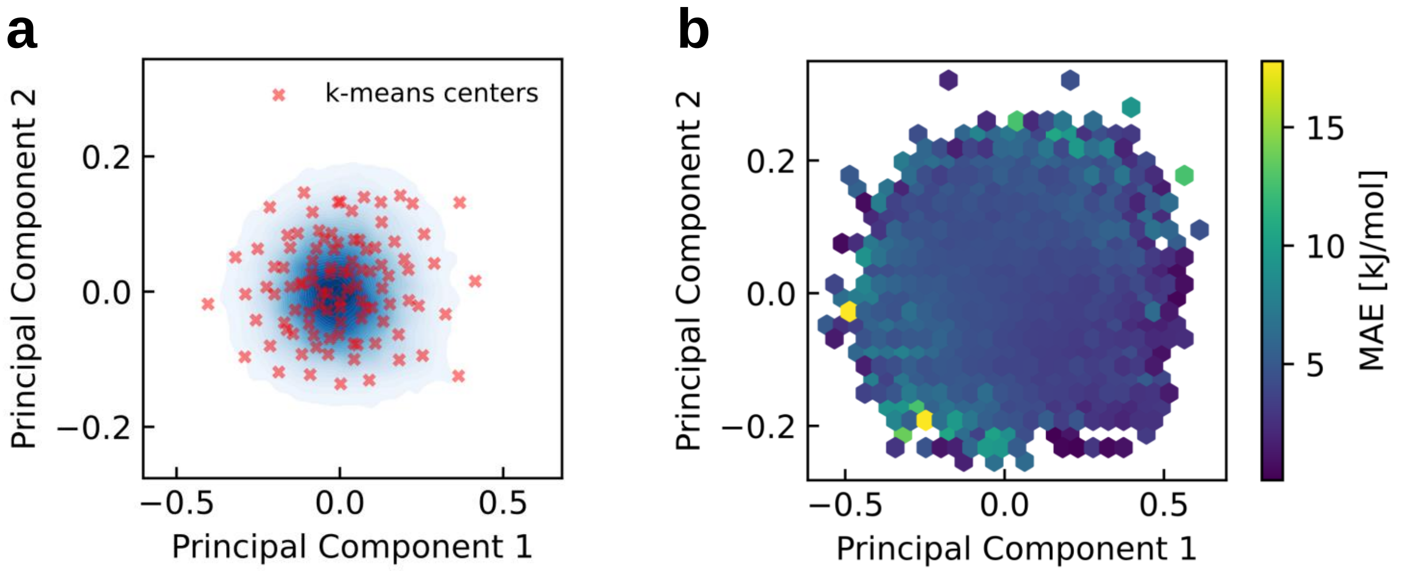

Supplementary Fig. 22 The accelerated model (detailed in Fig. 3) for the Al(Mg) alloy. (a) *k*-means centers plotted over a density-plot of the GB population in PCA space, and (b) a hexbin plot of the MAE across the PCA space.

SUPPLEMENTARY NOTE 3. SEGREGATION DATA ANALYSIS

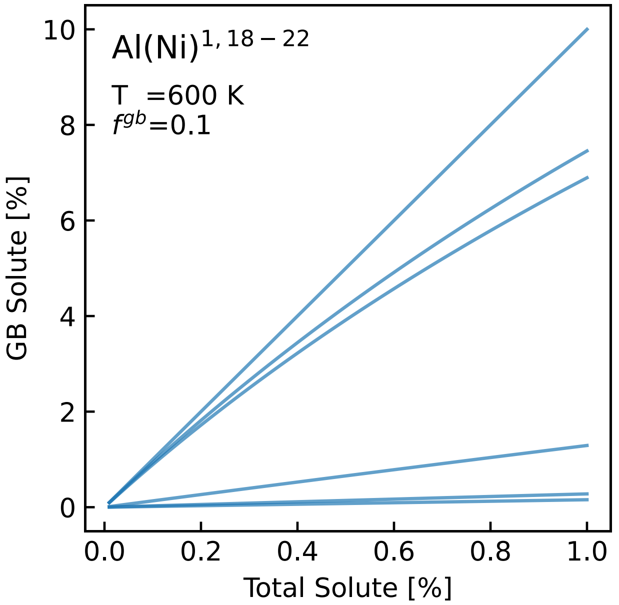

Supplementary Fig. 23 Predictions of GB solute segregation for the different Al(Ni)[1,18–22] Interatomic potentials in a polycrystal of average grain size 15 nm (f^{gb} ≈10%) at T = 600 K; even at a low total solute concentration of 1%, the predictions for X^{gb} can widely vary from 0.2% to 10%.

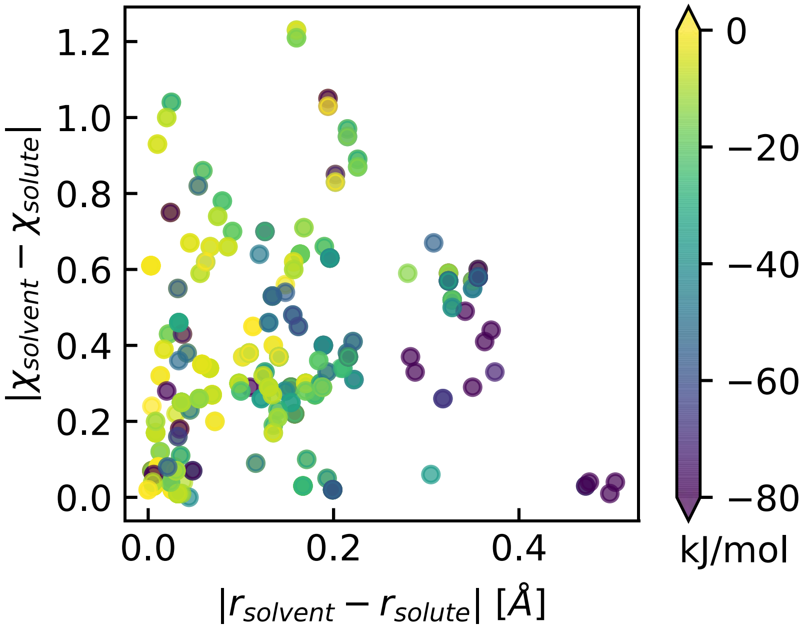

Supplementary Fig. 24 Predictions for the segregation tendency (25th percentile value) as a function of the difference in Pauling electronegativity (χ) and metallic radii (r).

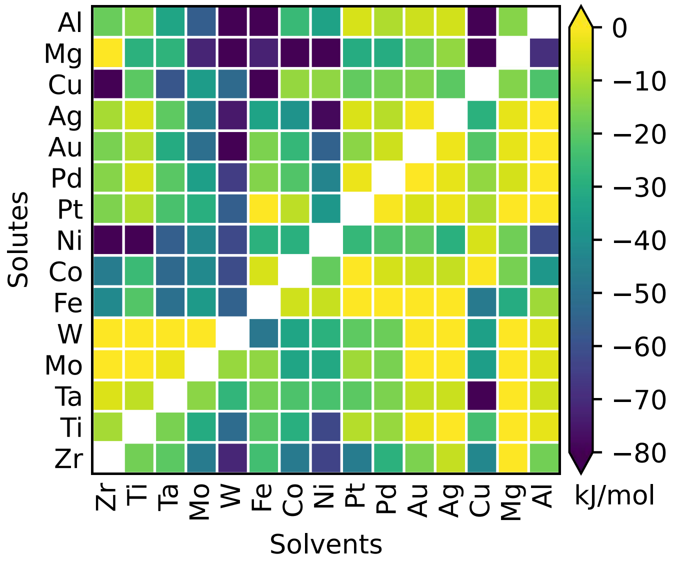

Supplementary Fig. 25 An alternate version of the Pettifor-type map in Fig.6. for the predicted segregation tendency across the alloy space, using interatomic potentials that has the most segregating spectra.

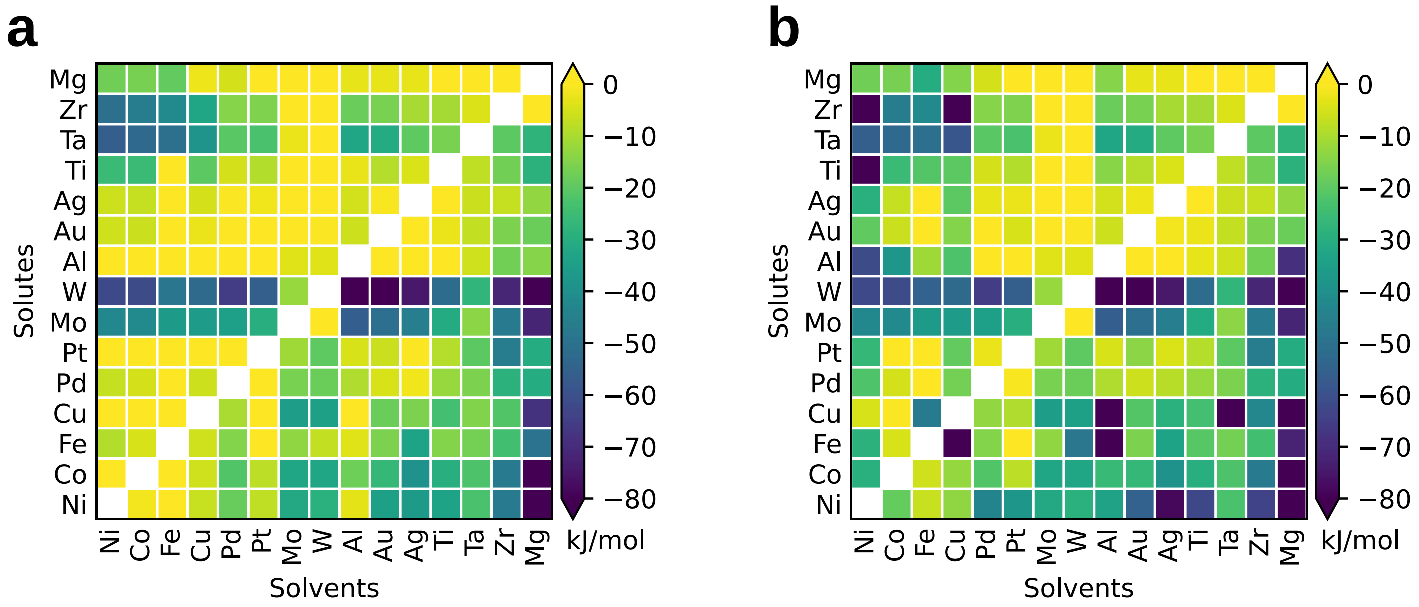

Supplementary Fig. 26 Predictions for the segregation tendency (quantified by the 25th percentile value) across the alloy space, with elements arranged by increasing metallic radii: Ni (smallest) → Mg (largest); for alloys with multiple potentials, we report (a) the least segregating spectra and (b) the most segregating spectra.

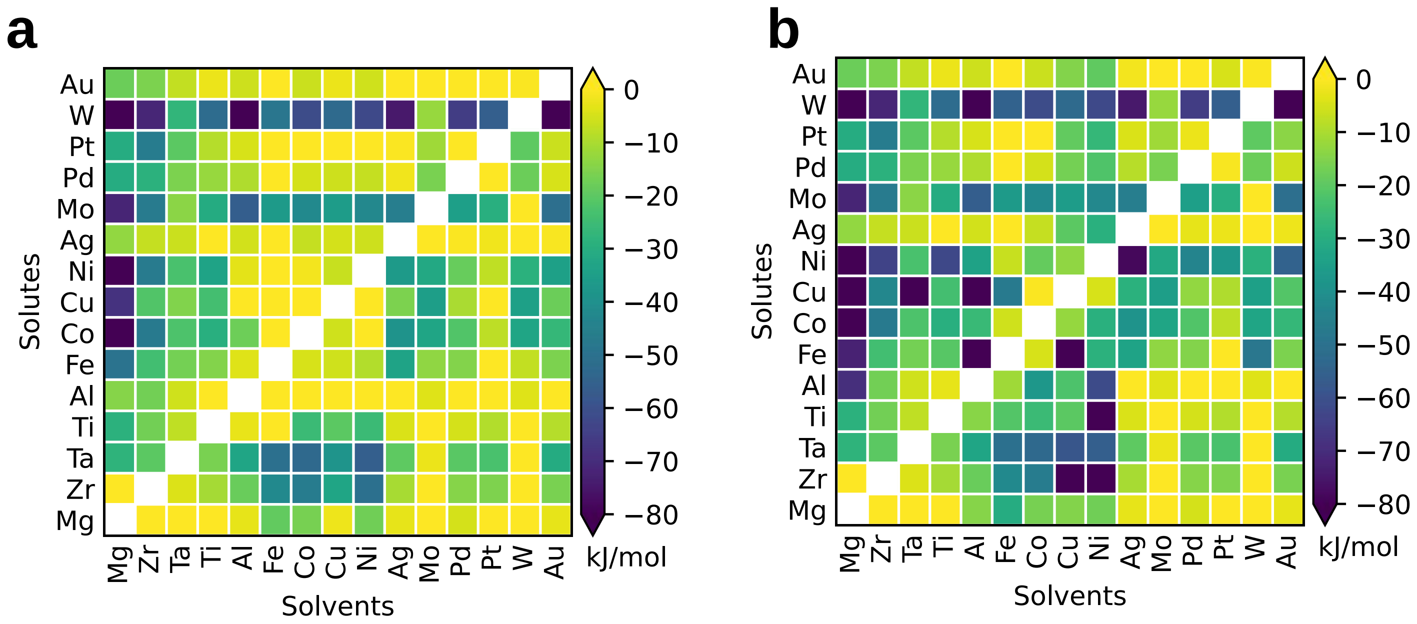

Supplementary Fig. 27 Predictions for the segregation tendency (25th percentile value) across the alloy space, with elements arranged by increasing Pauling electronegativity: Mg → Au; for alloys with multiple potentials, we report (a) the least segregating spectra and (b) the most segregating spectra.

SUPPLEMENTARY REFERENCES

1. Zhou, X. W., Johnson, R. A. & Wadley, H. N. G. Misfit-energy-increasing dislocations in vapor-deposited CoFe/NiFe multilayers. *Phys. Rev. B - Condens. Matter Mater. Phys.* **69**, 144113 (2004).
2. Foiles, S. M., Baskes, M. I. & Daw, M. S. Embedded-atom-method functions for the fcc metals Cu, Ag, Au, Ni, Pd, Pt, and their alloys. *Phys. Rev. B* **33**, 7983–7991 (1986).
3. Adams, J. B., Foiles, S. M. & Wolfer, W. G. Self-diffusion and impurity diffusion of fcc metals using the five-frequency model and the Embedded Atom Method. *J. Mater. Res.* **4**, 102–112 (1989).
4. Williams, P. L., Mishin, Y. & Hamilton, J. C. An embedded-atom potential for the Cu-Ag system. *Model. Simul. Mater. Sci. Eng.* **14**, 817–833 (2006).
5. Wu, H. H. & Trinkle, D. R. Cu/Ag EAM potential optimized for heteroepitaxial diffusion from ab initio data. *Comput. Mater. Sci.* **47**, 577–583 (2009).
6. Pan, Z., Borovikov, V., Mendelev, M. I. & Sansoz, F. Development of a semi-empirical potential for simulation of Ni solute segregation into grain boundaries in Ag. *Model. Simul. Mater. Sci. Eng.* **26**, 075004 (2018).
7. Purja Pun, G. P., Yamakov, V. & Mishin, Y. Interatomic potential for the ternary Ni-Al-Co system and application to atomistic modeling of the B2-L10 martensitic transformation. *Model. Simul. Mater. Sci. Eng.* **23**, 065006 (2015).
8. Liu, X. Y., Liu, C. L. & Borucki, L. J. New investigation of copper's role in enhancing Al-Cu interconnect electromigration resistance from an atomistic view. *Acta Mater.* **47**, 3227–3231 (1999).
9. Apostol, F. & Mishin, Y. Interatomic potential for the Al-Cu system. *Phys. Rev. B - Condens. Matter Mater. Phys.* **83**, 054116 (2011).
10. Jelinek, B. *et al.* Modified embedded atom method potential for Al, Si, Mg, Cu, and Fe alloys. *Phys. Rev. B - Condens. Matter Mater. Phys.* **85**, 245102 (2012).
11. Zhou, X. W., Ward, D. K. & Foster, M. E. An analytical bond-order potential for the aluminum copper binary system. *J. Alloys Compd.* **680**, 752–767 (2016).
12. Mendelev, M. I., Srolovitz, D. J., Ackland, G. J. & Han, S. Effect of Fe segregation on the migration of a non-symmetric $\sum 5$ tilt grain boundary in Al. *J. Mater. Res.* **20**, 208–218 (2005).
13. Liu, X. Y., Ohotnicky, P. P., Adams, J. B., Lane Rohrer, C. & Hyland, R. W. Anisotropic surface segregation in Al-Mg alloys. *Surf. Sci.* **373**, 357–370 (1997).
14. Liu, X. Y. & Adams, J. B. Grain-boundary segregation in Al-10%Mg alloys at hot working temperatures. *Acta Mater.* **46**, 3467–3476 (1998).
15. Mendelev, M. I., Asta, M., Rahman, M. J. & Hoyt, J. J. Development of interatomic potentials appropriate for simulation of solid–liquid interface properties in Al–Mg alloys. *Philos. Mag.* **89**, 3269–3285 (2009).
16. Dickel, D. E., Baskes, M. I., Aslam, I. & Barrett, C. D. New interatomic potential for Mg-Al-Zn alloys with specific application to dilute Mg-based alloys. *Model. Simul. Mater. Sci. Eng.* **26**, 045010 (2018).
17. Farkas, D. & Jones, C. Interatomic potentials for ternary Nb-Ti-Al alloys. *Model. Simul. Mater. Sci. Eng.* **4**, 23–32 (1996).
18. Angelo, J. E., Moody, N. R. & Baskes, M. I. Trapping of hydrogen to lattice defects in nickel. *Model. Simul. Mater. Sci. Eng.* **3**, 289–307 (1995).
19. Mishin, Y., Mehl, M. J. & Papaconstantopoulos, D. A. Embedded-atom potential for B2-NiAl. *Phys. Rev. B - Condens. Matter Mater. Phys.* **65**, 1–14 (2002).
20. Mishin, Y. Atomistic modeling of the γ and γ'-phases of the Ni-Al system. *Acta Mater.* **52**, 1451–1467 (2004).
21. Purja Pun, G. P. & Mishin, Y. Development of an interatomic potential for the Ni-Al system. *Philos. Mag.* **89**, 3245–3267 (2009).
22. Kumar, A. *et al.* Charge optimized many-body (COMB) potential for dynamical simulation of Ni-Al phases. *J. Phys.*

Condens. Matter **27**, 336302 (2015).

23. Landa, A. *et al.* Development of glue-type potentials for the Al-Pb system: phase diagram calculation. *Acta Mater.* **48**, 1753–1761 (2000).

24. Mendelev, M. I. *et al.* Development of interatomic potentials appropriate for simulation of devitrification of Al90Sm10 alloy. *Model. Simul. Mater. Sci. Eng.* **23**, 045013 (2015).

25. Zope, R. R. & Mishin, Y. Interatomic potentials for atomistic simulations of the Ti-Al system. *Phys. Rev. B - Condens. Matter Mater. Phys.* **68**, 024102 (2003).

26. O'Brien, C. J., Barr, C. M., Price, P. M., Hattar, K. & Foiles, S. M. Grain boundary phase transformations in PtAu and relevance to thermal stabilization of bulk nanocrystalline metals. *J. Mater. Sci.* **53**, 2911–2927 (2018).

27. Starikov, S. V., Lopanitsyna, N. Y., Smirnova, D. E. & Makarov, S. V. Atomistic simulation of Si-Au melt crystallization with novel interatomic potential. *Comput. Mater. Sci.* **142**, 303–311 (2018).

28. Farkas, D. & Caro, A. Model interatomic potentials and lattice strain in a high-entropy alloy. *J. Mater. Res.* **33**, 3218–3225 (2018).

29. Béland, L. K. *et al.* Features of primary damage by high energy displacement cascades in concentrated Ni-based alloys. *J. Appl. Phys.* **119**, 085901 (2016).

30. Stukowski, A., Sadigh, B., Erhart, P. & Caro, A. Efficient implementation of the concentration-dependent embedded atom method for molecular-dynamics and Monte-Carlo simulations. *Model. Simul. Mater. Sci. Eng.* **17**, 075005 (2009).

31. Bonny, G., Pasianot, R. C., Terentyev, D. & Malerba, L. Iron chromium potential to model high-chromium ferritic alloys. *Philos. Mag.* **91**, 1724–1746 (2011).

32. Zhou, X. W., Foster, M. E. & Sills, R. B. An Fe-Ni-Cr embedded atom method potential for austenitic and ferritic systems. *J. Comput. Chem.* **39**, 2420–2431 (2018).

33. Howells, C. A. & Mishin, Y. Angular-dependent interatomic potential for the binary Ni–Cr system. *Model. Simul. Mater. Sci. Eng.* **26**, 085008 (2018).

34. Bonny, G. *et al.* On the mobility of vacancy clusters in reduced activation steels: An atomistic study in the Fe-Cr-W model alloy. *J. Phys. Condens. Matter* **25**, 315401 (2013).

35. Bonny, G., Pasianot, R. C., Castin, N. & Malerba, L. Ternary Fe-Cu-Ni many-body potential to model reactor pressure vessel steels: First validation by simulated thermal annealing. *Philos. Mag.* **89**, 3531–3546 (2009).

36. Foiles, S. M. Calculation of the surface segregation of Ni-Cu alloys with the use of the embedded-atom method. *Phys. Rev. B* **32**, 7685–7693 (1985).

37. Onat, B. & Durukanoğlu, S. An optimized interatomic potential for Cu-Ni alloys with the embedded-atom method. *J. Phys. Condens. Matter* **26**, (2014).

38. Hoyt, J. J., Garvin, J. W., Webb, E. B. & Asta, M. An embedded atom method interatomic potential for the Cu-Pb system. *Model. Simul. Mater. Sci. Eng.* **11**, 287–299 (2003).

39. Purja Pun, G. P., Darling, K. A., Kecskes, L. J. & Mishin, Y. Angular-dependent interatomic potential for the Cu-Ta system and its application to structural stability of nano-crystalline alloys. *Acta Mater.* **100**, 377–391 (2015).

40. Mendelev, M. I., Sordelet, D. J. & Kramer, M. J. Using atomistic computer simulations to analyze x-ray diffraction data from metallic glasses. *J. Appl. Phys.* **102**, 043501 (2007).

41. Mendelev, M. I. *et al.* Development of suitable interatomic potentials for simulation of liquid and amorphous Cu-Zr alloys. *Philos. Mag.* **89**, 967–987 (2009).

42. Borovikov, V., Mendelev, M. I. & King, A. H. Effects of stable and unstable stacking fault energy on dislocation nucleation in nano-crystalline metals. *Model. Simul. Mater. Sci. Eng.* **24**, 085017 (2016).

43. Aslam, I. *et al.* Thermodynamic and kinetic behavior of low-alloy steels: An atomic level study using an Fe-Mn-Si-C modified embedded atom method (MEAM) potential. *Materialia* **8**, 100473 (2019).

44. Ackland, G. J., Mendelev, M. I., Srolovitz, D. J., Han, S. & Barashev, A. V. Development of an interatomic potential for phosphorus impurities in α-iron. *J. Phys. Condens. Matter* **16**, S2629 (2004).

45. Kim, H. K., Jung, W. S. & Lee, B. J. Modified embedded-atom method interatomic potentials for the Fe-Ti-C and Fe-Ti-N ternary systems. *Acta Mater.* **57**, 3140–3147 (2009).

46. Mendelev, M. I., Han, S., Son, W. J., Ackland, G. J. & Srolovitz, D. J. Simulation of the interaction between Fe impurities and point defects in V. *Phys. Rev. B - Condens. Matter Mater. Phys.* **76**, 214105 (2007).

47. Zhang, Y., Ashcraft, R., Mendelev, M. I., Wang, C. Z. & Kelton, K. F. Experimental and molecular dynamics simulation study of structure of liquid and amorphous Ni62Nb38 alloy. *J. Chem. Phys.* **145**, 204505 (2016).

48. Smirnova, D. E. & Starikov, S. V. An interatomic potential for simulation of Zr-Nb system. *Comput. Mater. Sci.* **129**, 259–272 (2017).

49. Bonny, G., Terentyev, D., Pasianot, R. C., Poncé, S. & Bakaev, A. Interatomic potential to study plasticity in stainless steels: The FeNiCr model alloy. *Model. Simul. Mater. Sci. Eng.* **19**, 085008 (2011).

50. Bonny, G., Castin, N. & Terentyev, D. Interatomic potential for studying ageing under irradiation in stainless steels: The FeNiCr model alloy. *Model. Simul. Mater. Sci. Eng.* **21**, 085004 (2013).

51. Béland, L. K. *et al.* Accurate classical short-range forces for the study of collision cascades in Fe–Ni–Cr. *Comput. Phys. Commun.* **219**, 11–19 (2017).

52. Samolyuk, G. D., Béland, L. K., Stocks, G. M. & Stoller, R. E. Electron-phonon coupling in Ni-based binary alloys with application to displacement cascade modeling. *J. Phys. Condens. Matter* **28**, (2016).

53. Maisel, S. B., Ko, W. S., Zhang, J. L., Grabowski, B. & Neugebauer, J. Thermomechanical response of NiTi shape-memory nanoprecipitates in TiV alloys. *Phys. Rev. Mater.* **1**, 33610 (2017).

54. Mendelev, M. I., Kramer, M. J., Hao, S. G., Ho, K. M. & Wang, C. Z. Development of interatomic potentials appropriate for simulation of liquid and glass properties of nizr2 alloy. *Philos. Mag.* **92**, 4454–4469 (2012).

55. Wilson, S. R. & Mendelev, M. I. Anisotropy of the solid-liquid interface properties of the Ni-Zr B33 phase from molecular dynamics simulation. *Philos. Mag.* **95**, 224–241 (2015).

56. Bonny, G., Bakaev, A., Terentyev, D. & Mastrikov, Y. A. Interatomic potential to study plastic deformation in tungsten-rhenium alloys. *J. Appl. Phys.* **121**, 165107 (2017).

57. Setyawan, W., Gao, N. & Kurtz, R. J. A tungsten-rhenium interatomic potential for point defect studies. *J. Appl. Phys.* **123**, 205102 (2018).
